# Electrochemical CO_2_ Reduction on Cu: Synthesis‐Controlled Structure Preference and Selectivity

**DOI:** 10.1002/advs.202101597

**Published:** 2021-10-23

**Authors:** Weiwei Quan, Yingbin Lin, Yongjin Luo, Yiyin Huang

**Affiliations:** ^1^ Fujian Provincial Key Laboratory of Quantum Manipulation and New Energy Materials College of Physics and Energy Fujian Normal University Fuzhou Fujian 350117 China; ^2^ Fujian Provincial Collaborative Innovation Center for Advanced High‐Field Superconducting Materials and Engineering Fuzhou 350117 China; ^3^ Fujian Key Laboratory of Pollution Control and Resource Reuse Fujian Normal University Fuzhou 350007 China

**Keywords:** catalysis, copper, electrochemical CO_2_ Reduction, structural engineering, synthesis

## Abstract

The electrochemical CO_2_ reduction reaction (ECO_2_RR) on Cu catalysts affords high‐value‐added products and is therefore of great practical significance. The outcome and kinetics of ECO_2_RR remain insufficient, requiring essentially the optimized structure design for the employed Cu catalyst, and also the fine synthesis controls. Herein, synthesis‐controlled structure preferences and the modulation of intermediate's interactions are considered to provide synthesis‐related insights on the design of Cu catalysts for selective ECO_2_RR. First, the origin of ECO_2_RR intermediate‐dominated selectivity is described. Advanced structural engineering approaches, involving alloy/compound formation, doping/defect introduction, and the use of specific crystal facets/amorphization, heterostructures, single‐atom catalysts, surface modification, and nano‐/microstructures, are then reviewed. In particular, these structural engineering approaches are discussed in association with diversified synthesis controls, and the modulation of intermediate generation, adsorption, reaction, and additional effects. The results pertaining to synthetic methodology‐controlled structural preferences and the correspondingly motivated selectivity are further summarized. Finally, the current opportunities and challenges of Cu catalyst fabrication for highly selective ECO_2_RR are discussed.

## Graphical abstract

This review summarizes the synthesis methodology‐controlled structure preference, and the structure engineering‐modulated intermediate generation, stabilization, and reaction on Cu‐based catalysts, toward highly selective electrochemical CO_2_ reduction to the products as‐desired.

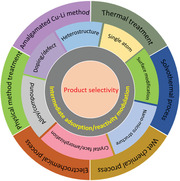

## 1 Introduction

The excessive combustion of fossil fuels and the massive emission of CO_2_ has caused serious energy and environmental problems and inspired the development of efficient CO_2_ recycle techniques.^[^ ^1^ ^]^ In this regard, the electrochemical CO_2_ reduction reaction (ECO_2_RR) holds great promise, as it allows one to convert CO_2_ into reusable carbon forms and can be further coupled with renewable energy sources (e.g., solar, wind, and tidal) for energy storage applications. In aqueous media, the ECO_2_RR can afford a wide range of C_1_ (e.g., HCOO^−^, CH_3_OH, CO, and CH_4_,), C_2_ (e.g., C_2_H_5_OH and C_2_H_4_) and C_2+_ (e.g., *n*‐C_3_H_7_OH) products.^[^ ^2^, ^3^ ^]^ However, ECO_2_RR selectivity tuning, especially toward the formation of high‐value‐added products, is hindered. This is because that ECO_2_RR generally involves multiple proton‐coupled electron transfer (PCET) with the difficulty of CO_2_ activation and reduction due to the high energy of its C═O bonds (750 kJ mol^−1^; cf. C—H (411 kJ mol^−1^) and C—O (327 kJ mol^−1^)).^[^ ^4^ ^]^ As Cu is the only metal promoting the formation of high‐order ECO_2_RR products (e.g., hydrocarbons and alcohols) at significant rates,^[^ ^5^, ^6^, ^7^, ^8^, ^9^ ^]^ the development of Cu‐based catalysts allowing one to overcome the high energy barriers of the ECO_2_RR and render it highly selective is a matter of great practical significance.

To date, the ECO_2_RR selectivities and product yields achieved over Cu catalysts remain unsuitable for practical applications.^[^ ^9^ ^]^ Typically, more than 16 individual C_1_—C_3_ products were produced over Cu catalysts in KHCO_3_ solution at −0.7 to −1.2 V versus RHE.^[^ ^2^ ^]^ As revealed by mechanistic studies at microscopic levels,^[^ ^3^ ^]^ the product selectivity of Cu catalysts is strongly correlated to the adsorption properties of intermediates such as *OCHO, *COOH, and *CO. However, Cu has moderate binding energies for most intermediates.^[^ ^10^ ^]^ Additionally, according to the Sabatier principle, the poor selectivity and activity of pure Cu catalysts are also due to the limitation of the linear scale relationship of adsorption energies between sequential reaction intermediates.^[^ ^11^ ^]^ Such limitations can be circumvented through structural engineering, which allows relatively independent modulation of some intermediates and thus enables reaction pathway/rate tuning to obtain the desired products at proper efficiencies. Based on this fact, numerous Cu‐based ECO_2_RR electrocatalysts structurally engineered at different scales have been developed over the past decade, as exemplified by Cu nanopolyhedra,^[^ ^12^, ^13^ ^]^ Cu nanowires,^[^ ^14^ ^]^ oxide‐containing Cu electrodes,^[^ ^15^ ^]^ roughened Cu surfaces,^[^ ^16^ ^]^ Cu surfaces modified by plasma treatment^[^ ^17^ ^]^ and electropolishing,^[^ ^18^ ^]^ and Cu‐based bimetallic catalysts.^[^ ^19^, ^20^ ^]^ However, the effects of structural engineering on the modulation of key intermediate generation, adsorption, and reactions are not yet fully understood, and the purposeful structural engineering of Cu catalysts therefore remains challenging.

Given that the choice of a proper synthetic method is of key importance for obtaining Cu catalysts with the desired structure, numerous such methods have been developed, e.g., those relying on pure metal melting, jet molding, mechanical alloying, composite explosive welding, electrochemical deposition and colloidal chemistry, impregnation, and hydrothermal processes.^[^ ^9^, ^15^, ^21^ ^]^ In many cases, different synthetic methods lead to different but unique atomic, nano‐, and/or microstructural characteristics. For example, electrodeposition based on a programmed square‐wave potential process has been used to prepare tetrahexahedral metal nanocrystals with abundant high‐index [310] facets.^[^ ^22^ ^]^ However, very few research reports and reviews have focused on the synthesis‐guided structure preference and selectivity. Hence, a review focusing on the use of synthesis‐directed structure design and intermediate modulation to obtain specific ECO_2_RR products could boost the development of highly selective Cu‐based catalysts.

The present review aims to provide a critical and timely understanding of synthetic methodology‐directed structural engineering and intermediate modulation, thus facilitating the design of Cu catalysts for highly selective and facile ECO_2_RR. In this review, we briefly introduce intermediate modulation and the key steps of the ECO_2_RR affording different products. Then we discuss the seven kinds of structural engineering based on diversified synthesis controls and further associate them with the modulation of intermediate generation, adsorption, and reaction as well as additional effects. Finally, we summarize the typical synthetic methodologies used to directly engineer catalyst structure and influence selectivity, and discuss the current opportunities and challenges of the design of efficient Cu‐based ECO_2_RR catalysts.

## 2 Mechanism of ECO_2_RR on Cu

**Figure 1:**
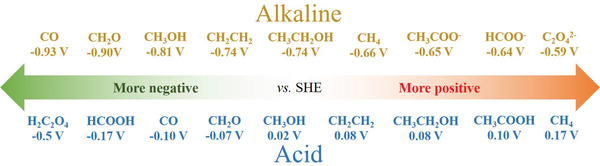
Standard reduction potentials (vs SHE) for ECO_2_RR to different products in alkaline and acidic solutions under the standard condition (25 °C and 1.0 atm).^[^ ^4^, ^26^ ^]^ The pH of acid and alkaline in the figure is 0 and 14, respectively.

As the CO_2_ molecule features two equivalent and stable C═O bonds with a length of 1.12 Å,^[^ ^23^ ^]^ relatively negative reduction potentials are usually required for the ECO_2_RR, which involves multiple PCET processes on most Cu catalysts. The common ECO_2_RR products formed on Cu are CO, CH_3_OH, CH_4_, HCOOH, C_2_H_5_OH, C_2_H_4_, H_2_C_2_O_4_, and so on. **Figure** **1** presents the corresponding standard thermodynamic reduction potentials (vs SHE), revealing that in alkaline media, they are generally more positive for the generation of C_2/2+_ products than for the formation of C_1_ products (except CH_4_). A similar trend is observed in acidic media, especially for C_2_H_5_OH and CH_3_COOH production. However, the generation of complex products (e.g., ethylene) normally involves multistep processes and features higher energy barriers and larger overpotentials compared to those for simple products, e.g., formic acid and CO.^[^ ^24^ ^]^ As a result, the potential region for the generation of C_2/2+_ products may overlap with that for the generation of C_1_ products, and both regions may also be close to that of hydrogen evolution (0 V).^[^ ^25^ ^]^ Therefore, the control of ECO_2_RR selectivity is difficult.

**Figure 2:**
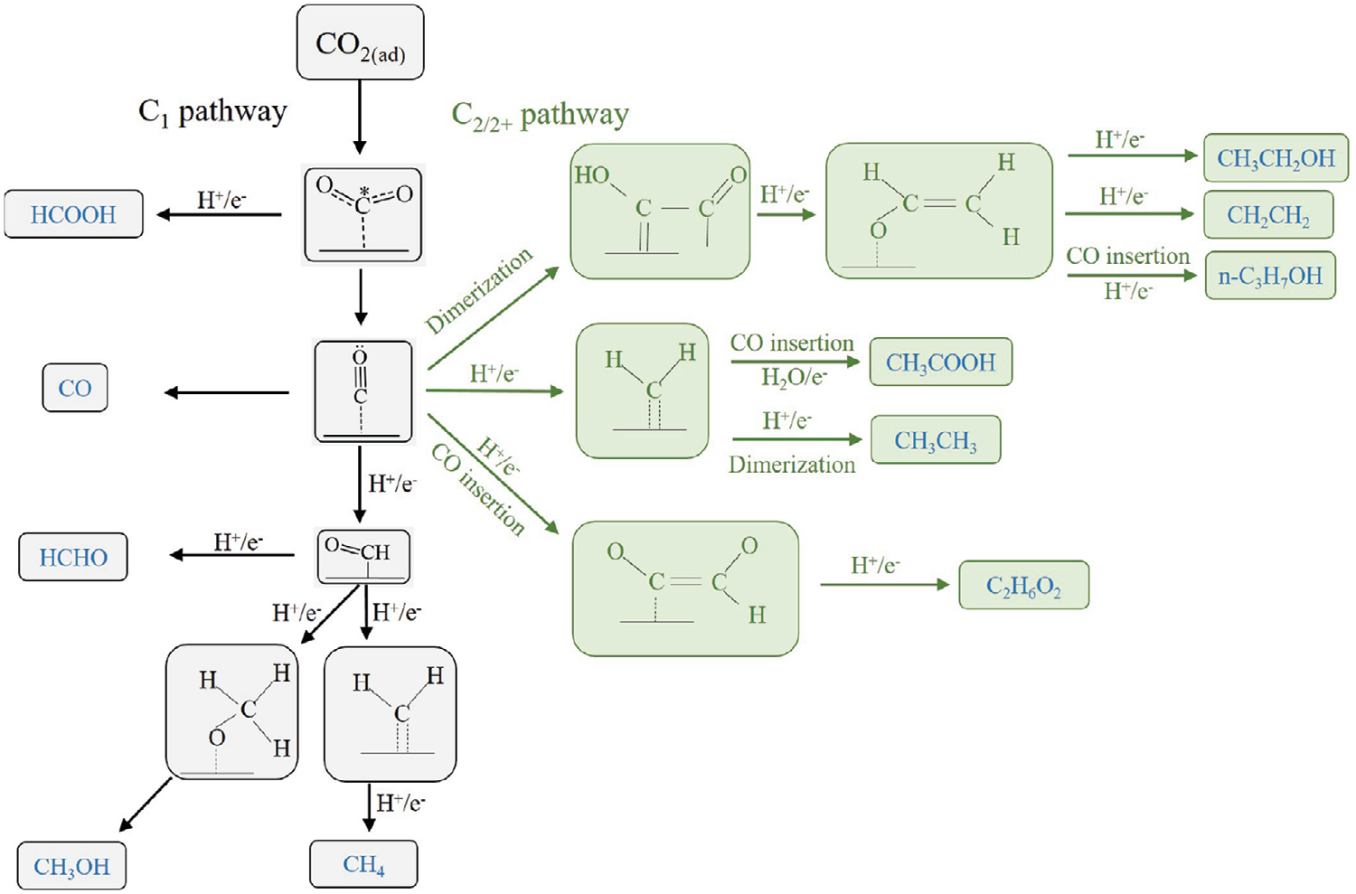
The general C_1_ and C_2/2+_ pathways on the Cu catalyst surface.

In general, Cu‐catalyzed ECO_2_RR involves three steps,^[^ ^4^ ^]^ namely, 1) reactant adsorption and interaction with the active sites on the catalyst surface, 2) multiple electron and/or proton transfer for reactant activation and reduction, and 3) the desorption of carbon‐based products and the recovery of the catalyst surface. The reactant mentioned above could be gaseous CO_2_ or equilibrium‐exchanged CO_2_ from HCO_3_^−^, as depicted in Equations (1) and (2).^[^ ^27^, ^28^ ^]^ Among these processes, the step of *CO_2_^•−^ generation via one‐electron transfer to the reactant may have a high energy barrier among the reaction steps, and is possibly rate‐limiting in many cases for ECO_2_RR (**Figure** **2**).^[^ ^29^, ^30^, ^31^ ^]^ More importantly, the adsorption strength of this intermediate may strongly influence the selectivity for primary products. As mentioned above, different metal and nonmetal catalysts give different ECO_2_RR products, ^[^ ^6^, ^7^, ^9^ ^]^ primarily because of their different adsorption abilities and reactivities for the intermediates, e.g., *CO_2_^•−^. Cu catalysts moderately strongly bind *CO_2_^•−^ and the deuterogenic *CO intermediates to generate diverse products (Figure 2), i.e., are intrinsically poorly selective. Notably, the modulation of Cu active sites for weakening *CO_2_^•−^ adsorption may lead to the formation of HCOOH, whereas the enhancement of this adsorption may facilitate the formation of *COOH and, ultimately, CO.^[^ ^4^ ^]^ Besides *CO_2_^•−^, *CO is another key intermediate for product selectivity modulation and can be further reduced to CH_4_ and CH_3_OH through successive protonation processes, the exact mechanisms of which depend on both thermodynamic and kinetic controls, and may involve the formation of *CHO as the rate‐limiting step.^[^ ^11^ ^]^

(1) $$CO_{2}+H_{2}O⇌\mathrm{HC}{O_{3}}^{-}+H^{+}\;\mathrm{pK}=6.35$$

(2) $$\mathrm{HC}{O_{3}}^{-}⇌{{\mathrm{CO}}_{3}}^{2-}+H^{+}\;\text{pK}=10.33$$

*CO is also viewed as the pivot intermediate for C–C coupling and C_2/2+_ product formation. Thus mechanistic studies can also adopt CO as the feedstock for electrochemical reduction to specific products with further identification of the produced intermediates.^[^ ^32^ ^]^ The detailed pathways of C–C coupling on Cu remain debated, with several widely accepted pathways presented in Figure 2.^[^ ^2^, ^24^, ^33^, ^34^ ^]^ The formation of C_2_H_4_ and C_2_H_5_OH is believed to involve carbene and CO insertion, *CO–COH coupling, and *CO dimerization. Among these pathways, that involving *CO dimerization is widely accepted, and this dimerization is also viewed as the rate‐limiting step for C_2/2+_ product formation.^[^ ^24^, ^33^ ^]^ Subsequent PCET affords *CH_2_CHO, which is then converted into either C_2_H_4_ or C_2_H_5_OH, depending on the CO—CO^−^ binding strength.^[^ ^2^ ^]^ The formation of *n*‐C_3_H_7_OH is believed to involve the coupling of C_1_ species (e.g., *CO) with adjacent C_2_ species (e.g., *CH_3_CHO and *CH_3_—CH), which requires a relatively high coverage of C_2_ intermediates that can be realized on atom‐ and structure‐optimized surfaces.^[^ ^2^ ^]^ The generation of C_2_H_6_ and CH_3_COOH is believed to involve *CH_2_ as the selectivity‐determining species, with subsequent hydrogenation and the coupling of the thus produced *CH_3_ affording C_2_H_6_, while the insertion of CO into *CH_2_ ultimately affords CH_3_COOH.^[^ ^2^ ^]^ During the formation of C_2_H_6_O_2_, *CHO produced via the protonation of *CO is further inserted into *CO to generate C_2_H_2_O_2_ and ultimately C_2_H_6_O_2_.

As mentioned above, Cu electrocatalysts feature moderate binding strengths for most C‐based intermediates and limited by scaling relationships, therefore exhibiting poor selectivity. Among the improved strategies, one can optimize intermediate stabilization via the modification of the catalyst surface with ligands, the alloying of Cu with other metals, and electronic structure tuning via promoter addition.^[^ ^35^ ^]^ Consequently, one can reduce the ECO_2_RR overpotential and improve selectivity by optimizing the adsorption energy of key intermediates. The design of Cu electrocatalysts based on kinetic property optimization can also circumvent the aforesaid limitations and allows one to achieve high selectivity. For example, Cu electrocatalysts with abundantly porous structures not only facilitate local pH control, but also promote the enrichment of key adsorbed intermediates such as *CO to increase the selectivity for high‐value‐added products.^[^ ^36^ ^]^ Therefore, the selectivity and activity of ECO_2_RR on Cu are influenced by both modulating intermediate‐catalyst interaction and the local reaction environment especially the pH and local reactant/intermediate concentration. A selectivity map for ECO_2_RR products painted by Pérez‐Ramírez and co‐workers via a modeling processing has demonstrated the critical impacts from the local reaction environment descriptors (local pH and CO_2_ concentration).^[^ ^37^ ^]^ To control these descriptors toward favorable ECO_2_RR, the liquid electrolyte flow cell and membrane electrode assembly (MEA) system have been widely used recently. Both the devices have the gas diffusion electrodes (GDE) (for MEA system the GDE is pressed up with ion exchange membrane),^[^ ^38^ ^]^ which can overcome the mass‐transfer resistance of CO_2_ and facilitate the accumulation of reactant/intermediate, thus promoting the reaction activity and tuning the selectivity.^[^ ^39^ ^]^ Additionally, the resultant high reaction rate would change the local pH environment,^[^ ^40^, ^41^ ^]^ further tuning the ECO_2_RR to the products as desired. Therefore, the liquid electrolyte flow cell and MEA system may assist the ECO_2_RR on the well‐designed Cu catalysts at high reaction rate and optimized selectivity, promoting its industrial application which requires the industrially relevant currents of 200 mA cm^−2^ or more.^[^ ^42^ ^]^

Overall, multiscale structural engineering in combined with modulation of local pH and reactant/intermediate concentration allows one to break the scaling relationships to optimize both selectivity and activity. In the subsequent sections, we discuss diverse structural engineering approaches in association with intermediate modulation and synthesis controls, and also highlight some synthesizing Cu‐based catalysts for high‐rate CO_2_ conversion.

## 3 Synthesis and Structural Engineering

### 3.1 Alloys and Compounds

The alloying of Cu with other metals or the formation of Cu compounds can be easily achieved via diverse synthetic approaches, which would further alter the electronic structure of Cu, thus tuning the adsorption and reactivity of key intermediates on Cu active sites. Herein, we discuss these two kinds of structural engineering from synthesis controls ultimately to intermediate modulation.

#### 3.1.1 Cu‐Based Alloys

Pd, Ag, and Au can be alloyed with Cu as secondary metals using wet chemical methods,^[^ ^43^, ^44^, ^45^ ^]^ with Pd receiving particularly much attention. Lu et al. prepared Pd*_x_* Cu*_y_* hydrogels via reduction with NaBH_4_ and dried them using supercritical CO_2_ to synthesize Pd*_x_* Cu*_y_* aerogels.^[^ ^30^ ^]^ The Pd_83_Cu_17_ aerogel achieved a current density of 31.8 mA cm^−2^ and a Faradaic efficiency (FE) of 80.0% for CH_3_OH generation at an overpotential of 0.24 V (vs Ag/Ag^+^). The synergistic effect between Pd and Cu facilitated the efficient adsorption of CO and CHO (which are the key intermediates for methanol production) on Cu^I^ + Cu^0^ surface species. Even for the same PdCu alloy, ECO_2_RR selectivity may depend on the wet chemical process used for synthesis, which highlights the need to consider other structural factors. For instance, spherical CuPd alloy nanoparticles prepared by a colloidal synthesis route using oleylamine as a solvent, surfactant, and reducing agent and Cu acetylacetonate and PdCl_2_ as metal precursors^[^ ^20^ ^]^ produced CO as the main ECO_2_RR product. This selectivity was ascribed to the increased energy of *H binding on the catalyst surface due to Pd addition. Therefore, the binding sites diversity and the coordination environment for intermediates were usually altered by alloying Cu with a second metal. In order to further promote the ECO_2_RR performance, recently, a polytetrafluoroethylene (PTFE) was used as the substrate for deposition of Cu and Ag via cosputtering processing to fabricate the GDE.^[^ ^46^ ^]^ Consequently, the Ag/Cu alloy phase catalyst exhibited high‐rate ethanol production in the flow cell test, with an FE of 41% and partial current of 250 mA cm^−2^ at −0.67 V versus RHE. These results indicate that alloying‐induced electronic structure modification allows one to modulate the adsorption strength of ECO_2_RR and hydrogen evolution reaction (HER) intermediates, combined with GDE to maximally alter the selectivity and activity of the former reaction.

A Sn/Cu electrocatalyst prepared by two‐step potentiostatic electrochemical deposition favored formate production.^[^ ^47^ ^]^ A high FE of 92% for formate production was achieved on the catalyst at the potential of −0.95 V versus RHE, with an impressing stability over the course of 12 h operation. It was revealed that the electronic regulation of Sn/Cu catalyst facilitates the two‐step reaction processes from CO_2_ to *CO_2_^−^ and further to HCOO*, which serves as the final intermediate for formate production. Besides Sn, In, Bi, and Sb can also be alloyed with Cu, exerting similar effects by suppressing the HER to promote ECO_2_RR performance.^[^ ^48^ ^]^ Non‐noble metals are normally alloyed with Cu via electrodeposition, which obviates the need to consider metal loss. In general, alloying can be an effective approach to tune the electronic structure of Cu (and, hence, the adsorption degrees of key intermediates) and can be combined with other effects to modulate ECO_2_RR selectivity and activity.

#### 3.1.2 Cu‐Based Compounds

Non‐metals can combine with Cu to form stable compounds, which allows one to tune the strength of intermediate binding on Cu–nonmetal compound catalysts. Yang prepared a novel CuSe nanocatalyst using a solvent coordination molecular template to produce methanol.^[^ ^49^ ^]^ During the synthesis of this electrocatalyst, Cu*_x_*—Se*_y_* nanoparticles were impregnated with NH_4_^+^‐coordinated Se to afford a template with protonated amine moieties and thus furnish a CuSe electrocatalyst that exhibited a new morphology and achieved a current density of 41.5 mA cm^−2^, an FE of 77.6%, and an overpotential of 285 mV for methanol production. The corresponding mechanistic study revealed that the intrinsic properties of CuSe make it sufficiently stable and facilitate the formation of *COOH and the adsorption of *CO, *CHO, *OCH_2_, and *OCH_3_, thus ultimately promoting the formation of methanol. Nonmetals can also be incorporated into Cu compounds using pyrolytic methods, as exemplified by the synthesis of Cu_3_P nanoparticles from NaH_2_PO_2_.^[^ ^50^ ^]^

**Figure 3:**
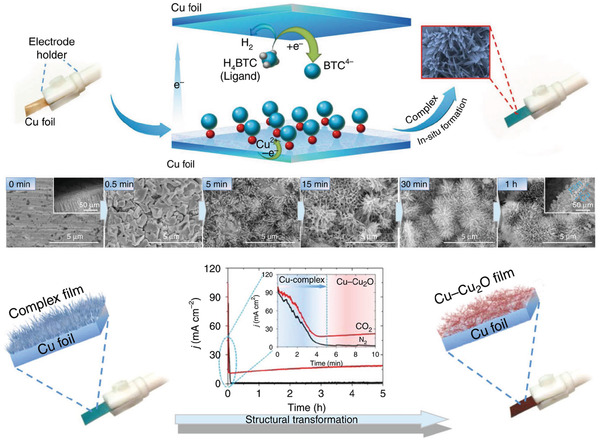
Electrodeposition synthesis of Cu complex on Cu foil and its SEM images at different synthesis time (H_4_BTC = pyromellic acid, BTC^4−^ = tetraanion of pyromellitic acid), as well as the electroreduction synthesis of Cu–Cu_2_O from this Cu complex at applied potential of −0.4 V versus RHE. Reproduced under the terms of the Creative Commons CC BY license.^[^ ^52^ ^]^ Copyright 2019, Nature Publishing Group.

Cu‐based oxides (e.g., Cu_4_O_3_) are a common type of Cu compounds exhibiting high ECO_2_RR activity^[^ ^51^ ^]^ and can be generated by the in situ reduction of Cu–organic frameworks to fabricate 3D structures.^[^ ^52^ ^]^ For example, a Cu‐complex film was prepared via the in situ electrooxidation of a Cu substrate and coordination with pyromellitic acid, with the subsequent in situ electroreduction of this film affording a Cu–Cu_2_O/Cu electrode (**Figure** **3**). During the ECO_2_RR test, the above electrode required overpotentials of 0.48 and 0.53 V for ethanol and acetic acid production, respectively, achieving an FE of 80% and a current density of 11.5 mA cm^−2^ for the formation of all C_2/2+_ products. In this case, CO_2_ activation and CO dimerization were enhanced because of the synergy between the active Cu^I^/Cu^0^ surfaces. Similar electrochemical reduction processes may also afford Cu nanoparticles, which largely favor ethylene production.^[^ ^53^ ^]^ Therefore, precursor properties play a key role in the electroreduction‐induced conversion into Cu oxides. Besides, these results suggest that the reconstruction of such precatalysts comprising Cu–organic frameworks or oxides (including bimetallic oxides^[^ ^54^ ^]^) may become decisive factors for excellent ECO_2_RR performance. Among the Cu‐based compounds, Cu oxides are particularly promising, as they can be synthesized by many methods (including electrochemical approach) and can maintain stable electrocatalytic performance. More importantly, compared to other Cu compounds (e.g., Cu_3_P and CuSe), the use of Cu oxides could avoid the introduction of heteroatoms to contaminate the electrolyte during the ECO_2_RR. Details on alloy and compound engineering are summarized in **Table** **1**.

**Table 1:** The key synthesis process, and structure–performance insights on various Cu‐based alloy and compound catalysts

Electrocatalysts	Synthesis method/process	Electrolyte and ECO_2_RR performance	Primary structure–performance relationship
CuPd nanoparticle alloy^[^ ^20^ ^]^	Colloidal synthesis route (300 °C)	0.1 m KHCO_3_ 87% CO FE at −0.9 V versus RHE 47 mA mg^−1^_Pd_	d‐band center of Cu upshift, increased energy barrier for *CO protonation and hydrogen evolution processes
Pd*_x_* Cu*_y_* aerogels^[^ ^30^ ^]^	Supercritical CO_2_ drying of Pd*_x_* Cu*_y_* hydrogels from wet chemical reduction	25 mol% [Bmim]BF_4_ + 75 mol% water 80.0% CH_3_OH FE at 0.24 V (vs Ag/Ag^+^) 31.8 mA cm^−2^	Synergistic effect from Pd/Cu facilitated adsorption of CO_ads_/CHO_ads_ on Cu(I) + Cu(0) species
CuAg alloy^[^ ^44^ ^]^	Physically melting method	0.05 m Cs_2_CO_3_ ≈35% FE for multicarbon oxygenates	Compressive strain on the surface alloy reduced coverage of adsorbed H and reduced oxophilicity
Cu_70_Ag_30_ thin film^[^ ^43^ ^]^	Physical vapor deposition	0.1 m KHCO_3_ Fourfold FE increase and threefold partial current density increase for liquid carbonyl compounds	Ag miscibility in Cu reduced the surface binding energies of oxygen‐containing intermediates
Ag/Cu alloy phase catalyst^[^ ^46^ ^]^	Physical cosputtering	1 m KOH in flow cell 41% ethanol FE at −0.67 V versus RHE 250 mA cm^−2^	Ag introduction to lower site‐carbon bonding ability and destabilize the unsaturated ethylene intermediates for ethanol production
AuCu nanoparticles^[^ ^55^ ^]^	Chemical reduction in organic solvent (200–280 °C)	0.1 m KHCO_3_ 80% CO FE at −0.85 V versus RHE	Formation of the three‐atoms‐thick gold overlayers with compressively strained properties
AuCu/Cu‐SCA catalyst ^[^ ^45^ ^]^	Electrodeposition	0.5 m KHCO_3_ 29% ethanol FE at −1.0 V versus RHE −5.59 mA cm^−2^	Au incorporation altered the binding energies of CH_2_CHO*, CH_3_CHO*, and CH_3_CH_2_O* for ethanol generation
Sn‐Cu bimetallic catalyst^[^ ^47^ ^]^	Two‐step electrodeposition	0.1 m KHCO_3_ 92% formate FE at −0.95 V versus RHE	Abundant interfaces and possible electron effect to promote CO_2_•^−^ and HCOO• generation
CuSe catalyst^[^ ^49^ ^]^	Solvothermal synthesis	[Bmim]PF_6_‐CH_3_CN‐H_2_O 77.6% methanol FE at −2.1 V (vs Ag/Ag^+^) 41.5 mA cm^−2^	Intrinsic properties of CuSe facilitated formation of *COOH, *CO, *CHO, *OCH_2_ and *OCH_3_
Cu_4_O_3_‐enrhched Cu catalyst ^[^ ^51^ ^]^	Solvothermal synthesis	0.5 m Cs_2_SO_4_ in flow cell 40% ethylene FE at −0.64 V versus RHE at 400 mA cm^−2^ (flow reactor)	Unknown
Cu_3_P/C catalyst ^[^ ^50^ ^]^	Annealing treatment	0.1 m NaHCO_3_ 47% CO FE at −0.3 V versus RHE	Unknown
Cu–Cu_2_O/Cu catalyst^[^ ^52^ ^]^	Electrodeposition of MOF followed by in situ electroreduction	0.1 m KCl 80% FE for C_2/2+_ product at −0.4 V versus RHE at 11.5 mA cm^−2^	Abundant surface active sites, suitable copper(I)/copper(0) ratio, and almost zero contacting resistance
Cu^II^/ade‐MOFs catalyst^[^ ^53^ ^]^	Wet chemical synthesis	0.1 m KHCO_3_ 73% hydrocarbon FE and 45% ethylene FE at a current of 8.5 mA cm^−2^ at −1.4 V versus RHE	Reconstruction of Cu^II^/ade‐MOFs and formation of Cu nanoparticles functionalized with N in ligand promoted the performance

### 3.2 Doping and Defects

The introduction of heteroatoms and defects allows one to effectively improve the intrinsic properties of Cu‐based ECO_2_RR electrocatalysts, e.g., via charge transfer between Cu and heteroatoms. Intermediate‐active site interactions may be affected by the modified electronic structure or the formation of extraordinary coordination chemistry, and the energy barrier of the rate‐limiting step can thus be reduced to favor the desired ECO_2_RR pathway. Shinagawa et al. prepared S‐doped Cu electrocatalysts^[^ ^56^ ^]^ by heating a mixture of Cu(NO_3_)_2_ with elemental S in ethylene glycol at 413 K for 12 h. Regardless of the initial S content, the catalysts underwent surface reconstruction and component reforming during the ECO_2_RR, which resulted in the formation of small particles of an S‐doped Cu electrocatalyst with a significantly low S content. This electrocatalyst exhibited a high FE of >80% for formate production at −0.8 V versus RHE, affording only traces of CO. The corresponding mechanistic study suggested that compared with the pure Cu catalyst, the S‐doped one probably featured a higher energy of *OCHO binding on surface sites due to the stabilizing effect of negatively charged S atoms and thus disfavored the *COOH and *CO pathways. The proposed surface configuration was predicted to be unfavorable for the adsorption of CO_2_ and *COOH because of the partial positive charge on their C atoms. Therefore, in both cases, the production of CO and the C_2/2+_ products derived from the same was disfavored.

O belongs to the same main group as S and may therefore play a similar role in Cu catalysts. Oxide‐derived Cu‐based electrocatalysts involve O modulation due to incomplete elimination. Eilert et al. found that the residual O in these catalysts is present as individual atoms rather than as a Cu‐bound (i.e., Cu oxide) form.^[^ ^57^ ^]^ The modification of Cu electronic structure by residual O increases the ability of active sites to bind *CO and thus increases *CO coverage to kinetically favor C–C coupling over hydrogenation processes and enhance ethylene production.

**Figure 4:**
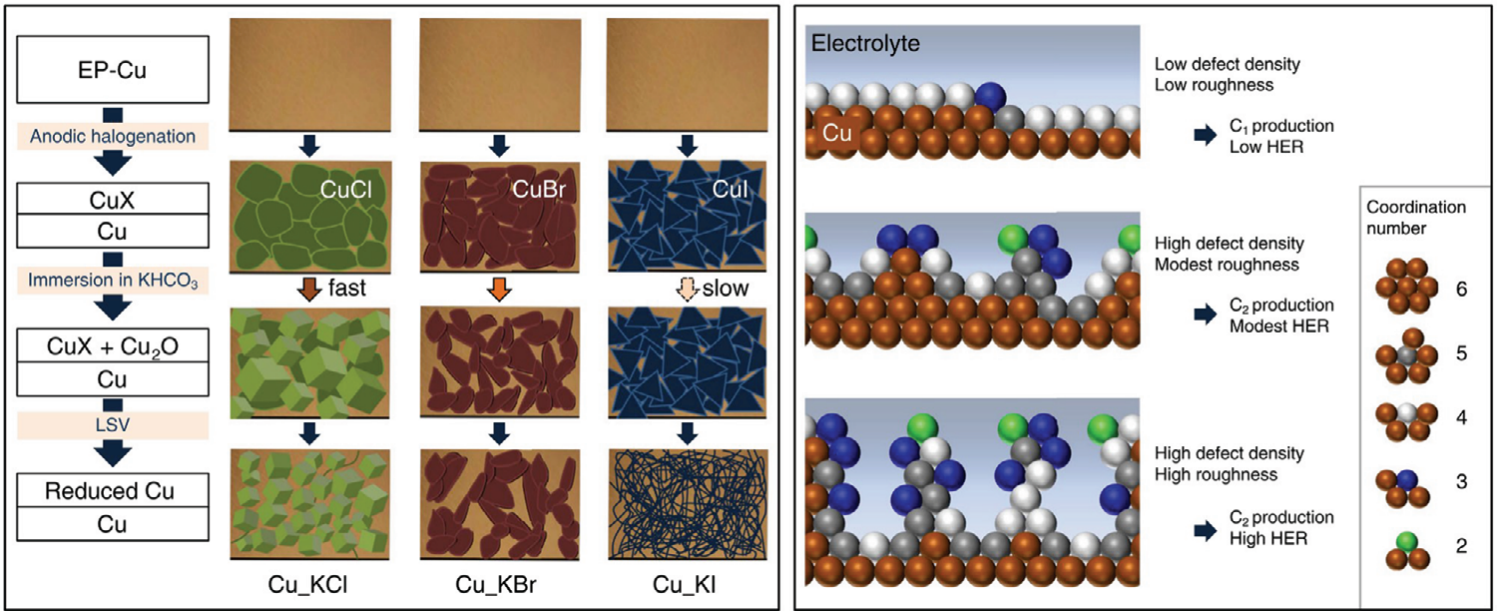
Schematics to show the morphology evolution of Cu catalysts prepared from different precursors, and defect structures‐influenced electrochemical selectivity. The sphere with green, blue, light gray, dark gray, and brown color represents the Cu atom with the coordination number of 2, 3, 4, 5, and 6, respectively. (EP‐Cu represents electropolished Cu foil.) Reproduced under the terms of the Creative Commons CC BY license.^[^ ^58^ ^]^ Copyright 2020, Nature Publishing Group.

Defect engineering such as the creation of atom vacancies allows one to adjust the electronic structure of Cu, enhance the electron‐capture ability of active sites, and alter the ECO_2_RR pathway. Kim and Palmore used anodic halogenation to convert the surface of electropolished Cu foils into CuCl, CuBr, or CuI (**Figure** **4**),^[^ ^58^ ^]^ with subsequent immersion into KHCO_3_ solution to form Cu_2_O and LSV treatment for the reduction of Cu_2_O to Cu affording Cu electrocatalysts with abundant surface defect sites and low roughness. Particularly, the Cu catalyst derived from CuCl showed uniformly sized cubic structures, with the lowest O and halogen contents. The Cu catalyst obtained from CuBr results in the wrinkled surface with shrinkage and some cracks. The Cu catalyst prepared from CuI causes a big morphological change during the LSV reduction process, with retaining the highest contents of O and halogen. In these cupric halide‐derived catalysts, defect sites (e.g., surface vacancies) as well as step sites and grain boundaries are highly coordination‐unsaturated entities and promote the formation of C_2/2+_ products. Consequently, the FE for C_2/2+_ products reached 70.7%, 71.5%, and 72.6% for CuCl‐, CuBr‐, and CuI‐derived Cu catalysts, respectively. Additionally, the low surface roughness of these catalysts was shown to suppress the HER and thus favor the ECO_2_RR (Figure 4). Vacancies, as the most common defect sites, can capture electrons in the metastable state to further inject them into CO_2_ and intermediates, and are commonly produced by plasma treatment. A plasma‐treated Cu nanocube electrocatalyst with a Cu(100) facet morphology^[^ ^59^ ^]^ achieved an FE of ≈73% for C_2/2+_ products at −1.0 V versus RHE, which was ascribed to the combined effects of defects and other factors (such as surface roughness and oxygen species in association with Cu^+^ species) on the binding of *CO to produce C_2/2+_ products. Although some roles of oxygen species in Cu‐based ECO_2_RR catalysts are still debatable, atom engineering has been widely proven to influence the properties of Cu‐based electrocatalysts and thus favor the ECO_2_RR. Structural engineering via doping and defect introduction shows high controllability, and could be proceeded after synthesis of specific structure types, providing variability for combination with other structural engineering methods. Details on doping and defect engineering are summarized in **Table** **2**.

**Table 2:** The key synthesis process, and structure–performance insights on various Cu‐based catalysts with doping and defect engineering

Electrocatalysts	Synthesis method/process	Electrolyte and ECO_2_RR performance	Primary structure–performance relationship
S‐doped Cu catalyst^[^ ^56^ ^]^	Solvothermal and electrochemical reduction	0.1 m KHCO_3_ 80% formate FE at −0.8 V versus RHE	S facilitated the *OCHO intermediate adsorption
O‐doped Cu catalyst^[^ ^57^ ^]^	Electrochemical reduction	4 × 10^−3^ m KCl in 0.1 m KHCO_3_ Ethylene production	O doping enhanced adsorption of *CO
Cu_4.16_CeO*_x_* catalyst^[^ ^60^ ^]^	Deposition‐participation process and annealing treatment	0.1 m KHCO_3_ 47.6% C_2_H_4_ FE and 62% C_2_H_4_–CH_4_ FE at −1.1 V versus RHE	Cuprous promoted the *CH_2_–*CH_2_ coupling and/or *C–*CO coupling, and *CH_2_ protonation
Cu catalyst^[^ ^58^ ^]^	Anodic halogenation and electroreduction processes	0.1 m KHCO_3_ 50.0% ethylene FE and 72.6% C_2/2+_ FE at −1.69 V versus RHE	Abundant surface defects and low roughness
Cu nanocube catalyst^[^ ^59^ ^]^	Electrochemically anodizing possessing and plasma treatments	0.1 m KHCO_3_ 73% C_2/2+_ FE at −1.0 V versus RHE	Defects, surface roughness, and oxygen species may alter the *CO adsorption strength

### 3.3 Crystal Facets and Amorphization

As the ECO_2_RR is a typical surface structure‐sensitive reaction, the surface atom arrangement of electrocatalysts, e.g., low‐index facets, stepped facets, out‐of‐plane sites, and amorphous surfaces, significantly affect selectivity and activity. Thus, surface‐guided synthesis is required for Cu catalysts.

#### 3.3.1 Low Index Facets

ECO_2_RR selectivity and activity are affected by the type of low‐index Cu facets, with orientation toward different crystal facets achieved by mechanical polishing together with electropolishing. Huang et al. used these processes to prepare single‐crystalline Cu(111), Cu(100), and Cu(110) surfaces,^[^ ^61^ ^]^ revealing that the Cu(100) surface featured the lowest overpotential for C_2_H_4_ production. This selectivity was ascribed to the maximized *CO coverage on this surface, which decreased the energy barrier for *CO dimerization, as demonstrated by density functional theory simulations. In addition to polishing‐based processing, square‐wave (SW) treatment also led to the oriented growth of (111) planes in the SW‐Cu_2_O/Cu film electrode.^[^ ^62^ ^]^ A KOH solution containing lactate ions was used as the electrolyte during electrochemical treatment, during which reduction and oxidation potentials were alternately applied to drive the conversion between Cu(OH)_2_^−^ (further to Cu_2_O and some CuO) and metallic Cu. To suppress HER occurrence and thus favor the ECO_2_RR, high pressure (60 atm CO_2_) was applied. As a result, the (111)‐oriented Cu film electrode achieved an FE of 98.2% for formate generation at −0.64 V versus RHE. Comparison of the energy barriers for the formation of carboxyl and formate intermediates (subsequently affording CO and formate, respectively) on the Cu(111) surface revealed that the latter barrier (10–20 kJ mol^−1^) was an order of magnitude lower than the former (100–350 kJ mol^−1^). Similarly, in situ electrochemical treatment during the ECO_2_RR was used to tune the Cu(100) facet‐oriented Cu electrocatalyst.^[^ ^19^ ^]^

Chemical synthesis in the presence of surfactants, e.g., hexamethylenetetramine and hexadecyltrimethylammonium bromide, has also been used to tune the crystal orientation of Cu catalysts and thus induce the formation of triangular 2D Cu nanosheets enclosed with two (111) planes.^[^ ^63^ ^]^ With the CO (key intermediate in ECO_2_RR) as the feedstock and use of a flow cell, the 2D Cu nanosheets could produce acetate at a partial current as high as 131 mA cm^−2^. On the other hand, in the presence of oleylamine, trioctylphosphine oxide, and CuBr, Cu nanocubes enclosed with abundant (100) planes were formed and exhibited an acetate FE of 48% at a partial current density of 131 mA cm^−2^ in alkaline solution. The enhanced generation of acetate was ascribed to the suppressed formation of other C_2/2+_ products due to the low exposure of planes ((110) and (100) ones) facilitating ethanol and ethylene generation. Similarly, octahedral Cu_2_O nanoparticles with (111) facets, cubic Cu_2_O nanoparticles with (100) facets, and truncated octahedral Cu_2_O nanoparticles with both (100) and (111) facets^[^ ^64^ ^]^ were prepared by wet chemical reduction and showed ethylene FEs of 45%, 38%, and 59%, respectively. Although chemical synthesis with facet control requires surfactants, it still offers the advantages of high scalability and controllability.

#### 3.3.2 Stepped Facets

**Figure 5:**
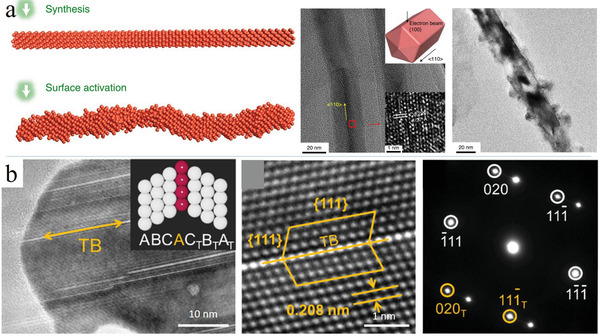
a) Schematic of the Cu nanowires with (100) surface, and the activated Cu nanowires after the electrochemical ECO_2_RR with surface steps. Adapted with permission.^[^ ^65^ ^]^ Copyright 2020, Nature Publishing Group. b) TEM image and the selected area electron diffraction of Cu twin boundary (TB, the white atoms are Cu(111) atoms and the red ones are TB atoms. The yellow circles are twin's spots and the white ones are matrix's diffraction spots). Adapted with permission.^[^ ^67^ ^]^ Copyright 2020, American Chemical Society.

Previous theoretical studies revealed that stepped facets are better active centers, as they more strongly bind key intermediates (e.g., *CO) and thus stabilize them for further dimerization.^[^ ^18^ ^]^ Choi et al. reported a wet chemical method employing CuCl_2_, glucose, and hexadecylamine as reactants to prepare Cu nanowires in aqueous solution (**Figure** **5**a).^[^ ^65^ ^]^ The obtained nanowires were subjected to 0.5 h electrochemical activation in 0.1 m KHCO_3_ at a reduction bias of −1.05 V, i.e., under conditions analogous to those of the ECO_2_RR test, to create stepped surfaces. The presence of steps on the nanowire surface was confirmed by high‐resolution scanning transmission electron microscopy (Figure 5a). An FE of 77.40% ± 3.16% was achieved for C_2_H_4_ production on Cu nanowires with highly stepped surfaces, and such performance could be maintained for ≈200 h in H cells. This high selectivity for ethylene was ascribed to the increased energy barrier for the C_1_ path (relative to that on the Cu(100) surface) and the high local coverage of 2*CO for C–C coupling.

High‐index facets comprising stepped surface structures can be obtained by physical vapor deposition. Typically, a Cu(751) thin film epitaxially grown on a Si(111) substrate was prepared and compared to the routinely grown Cu(111) and Cu(100) films on Al_2_O_3_ (0001) and Si(100).^[^ ^66^ ^]^ The Cu(751) facet was defined as the step notation of 4(110) × (311). Both Cu(100) and Cu(751) films showed increased selectivity for C_2/2+_ products, while the highest oxygenate‐to‐hydrocarbon ratio of 0.68 was obtained for the Cu(751) thin film electrode at −0.89 V versus RHE. The increased (compared to those of Cu(111) and Cu(100)) oxygenate selectivity of Cu(751) was ascribed to the lower number of nearest neighbors on the topmost layer of the Cu(751) film or the Cu(S) − [*n*(110) × (100)] surface and the resulting lower (compared to those of PCET processes) barriers for hydride transfer at a lower overpotential. In general, the engineering of stepped‐surface Cu catalysts is believed to enhance selectivity, reactivity, and even stability toward C_2/2+_ product formation. The synthesis chemistry should be guided to create stepped facets with abundant undercoordinated sites in simple but effective approaches.

#### 3.3.3 Out‐of‐Facet Sites

Out‐of‐facet sites referring to edges, corners, vertices, and twin boundaries, are often undercoordinated and have therefore received much attention.^[^ ^12^, ^14^, ^65^ ^]^ Hexarhombic docadehedron‐like, cube‐like, and octahedron‐like Cu nanocrystals were fabricated and investigated as ECO_2_RR catalysts.^[^ ^68^ ^]^ In particular, hexarhombic docadehedron‐like Cu nanocrystals achieved an FE of ≈25% with a partial current density of ≈3.5 mA cm^−2^ for ethanol production. The corresponding mechanistic study revealed that the edge sides (e.g., Cu(110)) lowered the stability of adsorbed *O to divert the ECO_2_RR pathway from ethylene to ethanol. Another computational work revealed the importance of the dual‐facet mechanism involving (110) edges and (100) terraces on cube‐like Cu nanocatalysts for ethylene production.^[^ ^13^ ^]^ Different morphologies lead to different levels of out‐of‐facet sites that can be controllably derived from different halide microcrystals,^[^ ^69^ ^]^ e.g., Cu nanofibers, nanodendrites, and nanocubes were generated from CuI, CuBr, and CuCl via electrochemical reduction, respectively. Cu nanofibers showed a high FE of 57.2% for C_2_—C_3_ products at −0.735 V versus RHE. In brief, these results suggested that out‐of‐facet sites have higher ECO_2_RR reactivity due to their high extent of coordinative unsaturation and may engage in synergistic interactions at intersection locations.

Twin boundaries and stacking faults are other attractive structures characteristic of Cu catalysts. Tang et al. prepared a Cu twin‐boundary catalyst (Figure 5b) using pulsed electrochemical deposition^[^ ^67^ ^]^ and revealed that the turnover frequency for CH_4_ generation on twin boundary atoms exceeded that observed for on‐plane atoms by three orders of magnitude. The twin boundary area can be obtained from twin boundary density, width (0.255 nm) and the electrochemical active area, then a local partial current density of 1294 mA cm^−2^ was calculated on twin boundaries, and the corresponding FE for CH_4_ generation was as high as 92%. The atoms on twin boundaries greatly enhanced the protonation of *CO, thus promoting its conversion to CH_4_. Similarly, a star decahedron Cu nanocatalyst with twin boundaries prepared by a wet chemical method lowered the formation energy of *CHO and thus facilitated the generation of CH_4_ (FE = 52.4% at −0.993 V).^[^ ^12^ ^]^ Fragmented Cu^[^ ^70^, ^71^ ^]^ and Cu assemblies^[^ ^72^ ^]^ are another kind of promising structures with a role similar to that of twin boundaries and can be easily obtained by electrochemical treatment or assembly, thus holding great promise for practical applications. In general, fine synthetic chemistry control is required to obtain twin boundaries or other types of out‐of‐facet sites.

#### 3.3.4 Amorphous Surfaces

Amorphous surfaces are special but common surface structures that do not have long‐range order (unlike their crystalline counterparts) but are rich in defects, dangling bonds, and specific localized electronic structures. Such unsaturated coordination properties may favor high reactivity and selectivity for the ECO_2_RR. Duan et al. used a weak reductant (tannic acid) at room temperature to prepare amorphous Cu nanoparticles^[^ ^21^ ^]^ and revealed that compared to crystalline Cu nanoparticles synthesized by NaBH_4_ reduction, the amorphous Cu nanoparticles showed better ECO_2_RR performance, exhibiting a total FE of 59% for the production of liquid fuels at −1.4 V and an FE of 22% for ethanol production. The enhanced selectivity and activity were ascribed to the stronger CO_2_ adsorption ability, larger electrochemical surface area, and fine particle size. The amorphization of Cu can also be achieved by exposure to air, as exemplified by the formation of Cu–In dendrites with a surface amorphous layer of ≈5 nm thickness.^[^ ^48^ ^]^ However, the widespread usage of amorphous Cu catalysts is hindered by their unclear structure–performance relationships and the difficulty of their repeatable synthesis. Atomic‐resolution operando characterization methods shed light on the action mechanism of these catalysts and their development direction. Details on crystal facet engineering and amorphization are summarized in **Table** **3**.

**Table 3:** The key synthesis process, and structure–performance insights on various Cu‐based catalysts with crystal and catalyst face engineering

Electrocatalysts	Synthesis method/process	Electrolyte and ECO_2_RR performance	Primary structure–performance relationship
SW‐Cu_2_O/Cu film cathode^[^ ^62^ ^]^	Square‐wave (SW) electrochemical redox cycling	0.5 m KHCO_3_ 98.2% formate FE at ‐0.64 V versus RHE (60 atm CO_2_)	Lower energy battier for formate intermediate formation on Cu(111)
(100)‐rich Cu catalyst^[^ ^19^ ^]^	In situ electrochemical treatment during ECO_2_RR	0.15 m KHCO_3_ in flow cell 90% C_2/2+_ FE at −0.67 V versus RHE 520 mA cm^−2^	High chemisorbed CO selectivity for further conversion of C2 products on Cu(100)
Triangular 2D Cu nanosheets^[^ ^63^ ^]^	Wet chemical reduction synthesis	2 m KOH 48% acetate FE at −0.74 V versus RHE 131 mA cm^−2^	Suppression of other C_2/2+_ products based on decreasing ratio of (110) and (100) planes
Truncated‐octahedral‐Cu_2_O nanoparticles^[^ ^64^ ^]^	Wet chemical reduction synthesis	0.5 m KHCO_3_ 59% ethylene FE at −1.1 V versus RHE 23.1 mA cm^−2^	Fermi level is lower on (111) than on (100) facets, enhancing charge transfer and promoting multielectron kinetics processes
A‐Cu nanowires^[^ ^65^ ^]^	Wet chemical synthesis of nanowires and electrochemical activation to form steps	0.1 m KHCO_3_ 77.4% ethylene FE at −1.01 V versus RHE 17.3 mA cm^−2^	High energy barrier C1 path and high local population of 2CO* intermediate
Cu (751) thin films^[^ ^66^ ^]^	Physical vapor deposition	0.1 m KHCO_3_ in flow cell 15% oxygenate FE and 51.7% hydrocarbon FE at −1.1 V versus RHE	Less nearest neighbors on the topmost layer of the Cu(751) film or the Cu(S)–[*n*(110) × (100)] surface
Cu nanofibers^[^ ^69^ ^]^	Electrochemically reduction of Cu halide	0.1 m KHCO_3_ 57.2% C2–C3 FE at −0.735 V versus RHE	Unknown
Hexarhombic docadehedron‐like Cu^[^ ^68^ ^]^	Wet chemical synthesis	0.1 m KHCO_3_ 25% ethanol FE at −1.2 V versus RHE ≈3.5 mA cm^−2^	Edge sides to lower the stability of adsorbed *O and tune ECO_2_RR pathway from ethylene to ethanol
Cu twin boundaries^[^ ^67^ ^]^	Pulsed electrochemical deposition	0.2 m KHCO_3_ 59% CH_4_ FE at −1.6 V versus RHE ≈7.04 mA cm^−2^	Twin boundaries enhanced protonation of adsorbed CO* to convert into CH_4_
Star decahedron Cu nanocatalyst^[^ ^12^ ^]^	Wet chemical synthesis	0.1 m KHCO_3_ 52.4% C_2_H_4_ FE at ≈−0.993 V versus RHE	Formation energy of *CHO intermediate was lowered on the twin boundaries
Cu overlayers on tetrahexahedral Pd nanocrystals ^[^ ^22^ ^]^	Underpotential deposition Cu on Pd(310)	0.1 m NaHCO_3_ ≈20% ethanol FE at ≈−0.46 V versus RHE	Unknown
Amorphous Cu nanoparticles^[^ ^21^ ^]^	Slow reduction by tannic acid at room temperature	0.1 m KHCO_3_ 59% liquid fuel FE and 22% ethanol FE at −1.4 V versus Ag/AgCl	Stronger CO_2_ adsorption ability, larger ECSA, and fine particle size

### 3.4 Heterostructures

Heterostructure synthesis normally involves the combination of two or more components, while heterostructure engineering enables the regulation of surface strain, electron transfer, and the synergistic effects of the two components based on the formation of strong interfacial coupling. In view of the above, heterostructure catalysts may exhibit unexpected reactivity and selectivity for the ECO_2_RR owing to their modified active sites with tunable electronic structures and the synergy on the interfaces.

#### 3.4.1 Cu–Noble Metal Heterostructures

As in the case of alloying, Cu–Ag, Cu–Au, and Cu–Pd systems are the most common Cu–noble metal heterostructures. Chem et al. used carbon paper as a support to prepare a Cu–Ag tandem catalyst via the simple physical mixing of Cu and Ag nanomaterial powders,^[^ ^73^ ^]^ showing that Ag was mainly responsible for the production of CO, while Cu was in charge of the subsequent C–C coupling of this intermediate transferred from Ag. Therefore, Cu–Ag heterostructures may also exhibit such tandem mechanisms by individually optimizing the different steps of the complicated ECO_2_RR and thus achieving a synergistic effect for C_2/2+_ product formation. Other heterostructure systems include amorphous CuO/crystalline Cu_2_O on AgCu foam,^[^ ^74^ ^]^ core–shell Ag@Cu heterostructures,^[^ ^75^ ^]^ and linker‐combined heterostructures.^[^ ^76^ ^]^ Fu et al. employed 4,4′‐bipyridine (bipy) as a linker to assemble Au nanoparticles on Cu nanowire surfaces and thus prepare an Au‐bipy‐Cu heterostructure with an FE of 25% for CH_3_CHO production.^[^ ^76^ ^]^ In this structure, Au promoted the production of CO, while Cu facilitated its C–C coupling, and bipy was responsible for the stabilization and protonation of *CO_2_. Generally, geometric effects and the tandem mechanism play more important roles in Cu‐based heterostructures than the electronic effect, as revealed by the systematic comparison of phase‐separated, ordered, and alloy CuPd catalysts.^[^ ^77^ ^]^ In highly efficient Cu–noble metal heterostructures, the noble metal is believed to promote the generation of *CO and thus increase the probability of its dimerization on Cu atoms adjacent to reaction sites.

#### 3.4.2 Cu–Non‐Noble Metal Heterostructures

Cu–non‐noble metal oxide/hydroxide heterostructures have received much attention in view of their low cost. One of the simplest of such catalysts, namely a Cu/Cu_2_O heterostructure, was prepared by Chang et al. via the electrochemical deposition‐induced introduction of Cu nanoparticles onto Cu_2_O films and exhibited notable methanol production activity.^[^ ^78^ ^]^ Electrochemical deposition was used to load Ce(OH)*_x_* on the Cu/PTFE GDE to prepare the heterostructure electrode,^[^ ^79^ ^]^ which showed a FE of 43% and the corresponding partial current as high as 128 mA cm^−2^ for ethanol production in the flow cell tests. The Cu/ZnO catalyst produced by the physical mixing of Cu and ZnO also promoted methanol formation because of the synergistic action (promotion of formate formation and its hydrogenation) of the two components at the interface.^[^ ^80^ ^]^ A ZnO‐shell/CuO‐core catalyst was prepared by wrapping CuO nanowires with ZnO via atomic layer deposition and promoted the formation of C_2/2+_ liquid fuels in an alkaline medium.^[^ ^81^ ^]^ The corresponding mechanistic study suggested that Zn‐modified Cu active sites could moderately bind CO and *CH_3_ to facilitate the formation of *COCH_3_, which acted as the key intermediate for ethanol generation. Main‐group metals (Sn, In) can also be used to form heterostructures with Cu. Typically, simple dipping and thermal decomposition processes were adopted to deposit In oxide on Cu(OH)_2_ nanowires, with subsequent in situ electrochemical reduction affording a Cu–In heterostructure catalyst^[^ ^82^ ^]^ that favored the production of CO by increasing the adsorption strength of *COOH and destabilizing *H adsorption on the interface. The stability of the Cu–In heterostructure catalyst under ECO_2_RR conditions could be further improved through the introduction of metastable nitrogen species (e.g., as in Cu_3_N).^[^ ^83^ ^]^

**Figure 6:**
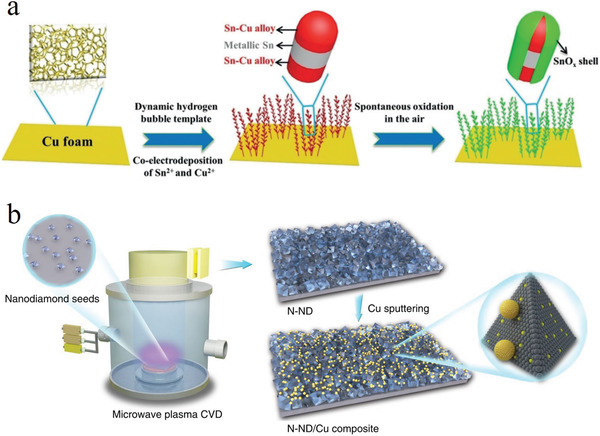
a) Schematic of the synthetic processes of 3D hierarchical Sn_2.7_Cu catalyst on Cu foam, and its high resolution transmission electron microscopy (HRTEM) image. Reproduced with permission.^[^ ^86^ ^]^ Copyright 2020, Wiley‐VCH. b) Schematic of preparation of N‐doped nanodiamond/Cu composite and its HRTEM image. Reproduced with permission.^[^ ^88^ ^]^ Copyright 2020, Nature Publishing Group.

As ECO_2_RR activity and selectivity are strongly related to the interface number/species and the overall structures of heterostructure catalysts, these aspects can be optimized in two ways. On the one hand, one can increase the number of heterostructure interfaces by optimizing the annealing temperature and atmosphere used to form nanoparticle heterostructures;^[^ ^84^ ^]^ besides, the formation of new interface species is also significant, e.g., new Cu–O–Mo sites in a Mo_8_–Cu heterostructure were shown to promote acetate production by facilitating the formation of *CH_3_.^[^ ^85^ ^]^ On the other hand, one can fabricate nanostructures for reactant/product transfer, as demonstrated by the 3D hierarchical Sn–Cu catalyst prepared by electrodeposition in the presence of evolved H_2_ bubbles (**Figure** **6**a).^[^ ^86^ ^]^ The favorable 3D structure and abundant interfaces of this catalyst lowered the energy barrier for the formation of *COOH, which resulted in an FE of 70% for formate production. Interestingly, the Cu–Sn heterostructure catalyst underwent surface reconstruction to form a Sn/SnO*_x_* interface (Figure 6a), which resulted in an even higher FE of ≈83.0% at −0.93 V versus RHE for formate production. The surface reconstruction was observed in the Cu/CuI heterostructure prepared by simple physically mixing,^[^ ^87^ ^]^ which results in lots of low‐coordinated sites, adsorbed Cu^+^ species and iodine on Cu surface. These favorable structure features induced stronger CO adsorption and thus promoted C–C coupling. With the utilization of flow cell, the Cu/CuI heterostructure catalyst reached an impressive C_2/2+_ partial current of 591 mA cm^−2^ at the potential of −1.0 V versus RHE. Generally, Zn, Mo, and Cu hold great promise as components of Cu–transition metal heterostructure catalysts for the production of C_2/2+_ products.

#### 3.4.3 Cu/Nonmetal Heterostructures

Nonmetal components such as graphene oxides,^[^ ^31^ ^]^ are usually used as supports for Cu‐based catalysts, and their effects on electrocatalytic ECO_2_RR performance are normally limited. However, the recent works on N‐doped nanodiamond–Cu heterostructures have challenged this viewpoint. Specifically, N‐doped nanodiamond films were prepared by microwave plasma‐enhanced chemical vapor deposition (CVD) (Figure 6b).^[^ ^88^ ^]^ Microwave irradiation provided a high‐temperature environment for CVD, while plasma treatment provided the basis for N doping, and the heterostructure was fabricated by Cu sputtering. The introduction of controllable amounts of N increased catalyst conductivity and influenced catalytic activity, while C enhanced the binding of *CO to increase its coverage and thus favor *CO–*CO coupling. Besides, the Cu—C bonds stably formed on the interface increased its stability in the heterostructure. As a result, the synthesized electrocatalyst showed an FE of ≈63% for the production of C_2_ oxygenates at −0.5 V versus RHE and exhibited a performance decay of only 19% after 120 h operation, which was indicative of high stability. This work has inspired theoretical and experimental research on heteroatom (e.g., B)‐doped nanodiamond and suggested that nonmetal phases with high chemical stability, surface area, conductivity, and electroactivity may be promising secondary phases for combination with Cu to form ECO_2_RR heterostructure catalysts. Details on heterostructure engineering are summarized in **Table** **4**.

**Table 4:** The key synthesis process, and structure–performance insights on various Cu‐based catalysts with heterostructure engineering

Electrocatalysts	Synthesis method/process	Electrolyte and ECO_2_RR performance	Primary structure–performance relationship
Cu–Ag tandem catalyst^[^ ^73^ ^]^	Physical mixing	1 m KOH in flow cell C_2/2+_ product partial current of 160 mA cm^−2^	Ag was responsible for CO production, and Cu was in charge of subsequent C–C coupling
Ag_15_Cu_85_ catalyst^[^ ^74^ ^]^	Additive‐assisted electrodeposition	0.5 m KHCO_3_ 33.7% ethanol FE at −1.0 V versus RHE; 6.9% n‐propanol FE at −0.9 V versus RHE	Ag facilitated CO generation, which was transferred to Cu surface for C–C coupling
Ag–Cu_2_O electrode^[^ ^89^ ^]^	Electrochemical deposition	0.2 m KCl 34.2% ethanol FE at −1.2 V versus RHE	Ag incorporation promoted CO production, and suppressed HER
Ag@Cu‐20 catalyst ^[^ ^75^ ^]^	Wet chemical synthesis (polyol method)	0.1 m KHCO_3_ 28.6% ethylene FE at −1.06 V versus RHE	Geometric effect and synergistic effect of Ag to produce CO and Cu to couple CO intermediate
Au‐bipy‐Cu catalyst^[^ ^76^ ^]^	Wet chemical synthesis	0.1 m KHCO_3_ 25% FE for CH_3_CHO production	Au promoted the CO production, and bipy could stabilize and make protonation of CO_2_*
Phase‐separated CuPd catalyst^[^ ^77^ ^]^	Two‐step wet chemical synthesis	1 m KOH in flow cell 63% C_2/2+_ FE	Geometric effect and synergistic effect of Pd to produce C1 intermediate and Cu for C–C coupling
Cu/Cu_2_O catalyst ^[^ ^78^ ^]^	Electrochemical deposition	0.1 m KHCO_3_ 53.6% methanol FE at −0.7 V versus RHE with 1.3 mA cm^−2^ (photoanode is TiO_2_)	Interface balanced the adsorption between *H and *CO intermediates
Cu/CuI catalyst^[^ ^87^ ^]^	Physical mixing	1 m KOH in flow cell 87% C_2/2+_ FE at −0.87 V versus RHE with partial current of 591 mA cm^−2^	Surface reconstruction to induce formation of Cu^+^ species and adsorbed iodine for enhanced CO adsorption
ZnO‐shell/CuO‐core bimetal‐oxide^[^ ^81^ ^]^	Atomic layer deposition of ZnO on CuO	1 m KOH in flow cell 48.6% C_2/2+_ FE at −0.68 V versus RHE with partial current of −97 mA cm^−2^	Zn‐modified Cu sites could moderately bind CO and *CH_3_ to promote *COCH_3_ formation
Cu–In heterostructure catalyst^[^ ^82^ ^]^	Dipping and thermal decomposition	0.1 m KHCO_3_ 93% CO FE at −0.6≈‐0.8 V versus RHE	Enhanced *COOH adsorption and *H destabilization adsorption on the interface
In_2_O_3_/Cu_3_N catalyst ^[^ ^83^ ^]^	Calcination of In(OH)_3_ supported on Cu_3_N	0.1 m KHCO_3_ 80% CO FE at −0.5 V versus RHE	Cu–In interface promotion effects
CuSn NPs/C‐A catalyst ^[^ ^84^ ^]^	Annealed in 5% H_2_ +95% N_2_ at 250 °C	0.1 m KHCO_3_ 80% HCOOH FE at −1.0 V versus RHE	Decreased Δ*G* for forming COOH* at the interface
Sn–Cu/SnO*_x_* Catalyst^[^ ^86^ ^]^	In situ electroreduction for formation of Sn/SnO*_x_* interface	1 m KOH 83.0% formate FE at −0.93 V versus RHE	In situ formed Sn/SnO*_x_* interface with optimal binding of HCOO* and suppressing HER
Mo_8_/Cu heterostructure^[^ ^85^ ^]^	Electrochemical deposition	NaHCO_3_ (unknown concentration) 48.68% acetate FE at −1.13 V versus RHE ≈110 mA cm^−2^	New Cu–O–Mo sites promote the *CH_3_ formation and can be coupling by CO_2_
N‐doped nanodiamonds/Cu heterostructure^[^ ^88^ ^]^	Chemical vapor deposition of NC and sputtering Cu	0.5 m KHCO_3_ ≈63% C2 oxygenates FE at −0.5 V versus RHE	N increased conductivity and tune Cu activity, C enhanced binding strength of *CO, stable Cu—C bond enhanced the stability
SnO_2_/CuO nanowires^[^ ^90^ ^]^	Atomic layer deposition	0.1 m NaHCO_3_ 13.4 CO FE with the solar‐to‐fuel efficiency of 14.4% under AM 1.5G illumination	Unknown
Au/Cu electrocatalyst^[^ ^91^ ^]^	Physical vapor deposition	0.1 m KHCO_3_ 5.8% ethanol FE and 4.7% n‐propanol FE at −0.97 V versus RHE with the current of 0.49 and 0.39 mA cm^−2^	Au promoted production of CO which was transferred to the nearby Cu for further C–C coupling; Locally alkaline environment
Ce(OH)*_x_*‐doped‐Cu catalyst^[^ ^79^ ^]^	Electrochemical deposition	1 m KOH in flow cell 43% ethanol FE at −0.7 V versus RHE with partial current of 128 mA cm^−2^	Adsorbed hydrogen hydrogenated surface *HCCOH for formation of ethanol

### 3.5 Single‐Atom Catalysts

As discussed above, the outcome of electrocatalytic CO_2_ reduction is primarily determined by the active sites of catalyst surfaces. Single‐atom Cu catalysts not only enable the complete exposure of highly active Cu atom sites to maximize atomic utilization, but also achieve uniform and adjustable Cu atom coordination status on the catalyst to afford an optimal electronic structure. Polymers, carbon, and metal compounds are usually used as substrates in the synthesis of such catalysts to anchor and stabilize single Cu atoms. Among them, carbon has received particular attention. Currently, single‐atom Cu catalysts face two significant challenges, namely those pertaining to catalyst stability and cooperativity with single‐atom Cu for multiple electron transfer processes to promote the production of CH_4_ and C_2/2+_ products.

In view of its high affinity to Cu, N is commonly introduced into carbon matrices. Yang et al. reported a high‐temperature gas‐transport approach of directly converting bulk Cu_2_O into a single‐atom Cu catalyst based on the strong interaction between Cu and N moieties^[^ ^92^ ^]^ and realized the scalable preparation of a single‐atom Cu‐modified carbon membrane catalyst for the ECO_2_RR.^[^ ^93^ ^]^ The above preparation involved the acquisition of Cu/ZIF‐8 nanoparticles, the mixing of polyacrylonitrile and a Cu/ZIF‐8 suspension, and polymer fiber production (via electrospinning) and pyrolysis. Single Cu atoms were found to strongly bind *CO, facilitating its successive hydrogenation to produce methanol. To promote the production of C_2/2+_ products on single‐atom Cu, one should ensure that the distance between the adjacent Cu sites is sufficiently small for effective C–C coupling, which requires fine synthesis controls. The distance between Cu–N*_x_* sites can be tuned by choosing an appropriate Cu content,^[^ ^94^ ^]^ e.g., at a Cu content of 4.9 mol%, C–C coupling occurred, leading to the production of C_2_H_4_ at neighboring Cu–N_2_ sites. Other configurations such as isolated or neighboring Cu–N_4_ and isolated Cu–N_2_ moieties and the low content of Cu (2.4 mol%) could only catalyze the formation of CH_4_. Thus, one can conclude that both distance and active site configuration play an essential role in determining product selectivity. Similarly, a Cu dimer embedded in a C_2_N monolayer was simulated to confirm the possibility of C_2_H_4_ production.^[^ ^95^ ^]^ Beyond these Cu–Cu synergies, another type of synergy is that between Cu and the adjacent C to generate C_2/2+_ products.^[^ ^96^ ^]^

**Figure 7:**
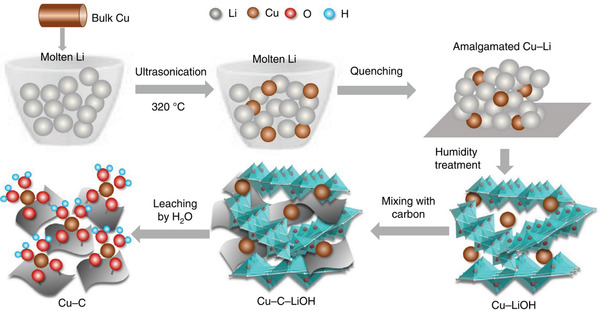
Schematic of synthesis of the carbon‐supported Cu single atom catalyst via the amalgamated Cu–Li method. Reproduced with permission.^[^ ^97^ ^]^ Copyright 2020, Nature Publishing Group.

A carbon‐supported Cu single‐atom catalyst was prepared using a Cu–Li amalgam (**Figure** **7**).^[^ ^97^ ^]^ Specifically, bulk Cu was dissolved in molten Li, and the melt was quenched and exposed to wet air to form a Cu–LiOH mixture. Finally, Cu–LiOH was blended with active carbon, and LiOH was removed by water to form the carbon‐supported single‐atom Cu catalyst. This new method holds great promise, as it can be extended to the large‐scale preparation of other single‐atom catalysts. Operando XAS analysis indicated that this single‐atom Cu (without potential application) could be converted into Cu*_n_* clusters (reduction potentials) modulated by hydroxyl groups on the carbon surface. These Cu*_n_* clusters are expected to enable C–C coupling via the *HCOO and *CO mechanisms to ultimately produce ethanol. As a result, the single‐atom Cu catalyst showed an FE of ≈91% at −0.7 V for ethanol production, with an onset potential of −0.4 V. Such interesting dynamical and reversible interconversion between single‐atom Cu and Cu*_n_* cluster nanoparticles was also observed by other operando techniques.^[^ ^97^, ^98^, ^99^ ^]^ These results strongly suggest the necessity of using operando techniques to uncover the real active sites and action mechanism of some single‐atom Cu catalysts.

In addition to carbon‐based substrates, metal‐based ones can also anchor single‐atom Cu by alloying or defect trapping. Jiao et al. prepared a Cu–Pd_10_Te_3_ catalyst via the stirring‐induced mixing of H_2_PdCl_4_, Cu(NO_3_)_2_, and Te nanowires.^[^ ^100^ ^]^ The obtained material featured Cu_1_^0^–Cu_1_ *^x^* ^+^ pairs on its surface, as follows from the results of X‐ray absorption fine structure spectroscopy experiments and theoretical calculations. The Cu_1_ *^x^* ^+^ moiety facilitated the adsorption of H_2_O molecules, which, in turn, stabilized CO_2_ on the Cu_1_^0^ moiety and thus promoted its activation. As a result, the Cu–Pd_10_Te_3_ catalyst delivered an FE of 92% for CO formation at −0.78 V versus RHE, completely suppressing the HER. Additionally, Cu_1_^0^–Cu_1_ *^x^* ^+^ pairs could be stabilized by catalyst surface defects. The surface defect trapping‐based synthesis of single‐atom Cu catalysts was demonstrated by the preparation of a Cu–CeO_2_‐4% system via hydrothermal and wet impregnation processes.^[^ ^29^ ^]^ This approach was based on the effective formation of Cu^2+^–oxygen vacancy pairs to maintain charge balance. Details on single‐atom engineering are summarized in **Table** **5**.

**Table 5:** The key synthesis process, and structure–performance insights on various Cu‐based catalysts with single‐atom engineering

Electrocatalysts	Synthesis method/process	Electrolyte and ECO_2_RR performance	Primary structure–performance relationship
CuSAs/TCNFs catalyst ^[^ ^93^ ^]^	Electrostatic spinning and pyrolysis	0.1 m KHCO_3_ 44% methanol FE at −0.9 V versus RHE, C1 product partial current: 93 mA cm^−2^	Strongly binding with *CO intermediate on single Cu
Cu–N–C‐800 catalyst ^[^ ^94^ ^]^	Pyrolysis	0.1 m KHCO_3_ 24.8% C_2_H_4_ FE and 13.9% CH_4_ FE at −1.4 V versus RHE, partial current: 6.84 and 3.83 mA·cm^−2^	Adjacent Cu–N_2_ moieties for *CO protonation and C–C coupling
Cu–C_3_N_4_ catalyst ^[^ ^96^ ^]^	Thermal treatment synthesis	0.1 m KHCO_3_ ≈5% ethanol FE at −1.6 V versus Ag/AgCl	Synergy between Cu for C‐terminated adsorption, and neighboring C for O‐terminated adsorption
Cu/C‐0.4 catalyst ^[^ ^97^ ^]^	Amalgamated Cu–Li method	0.1 m KHCO_3_ ≈91% ethanol FE at −0.7 V versus RHE	Dynamical formation of Cu_n_ cluster favorable for C–C coupling
Cu–N–C catalyst ^[^ ^98^ ^]^	Ball milling and pyrolysis	0.1 m CsHCO_3_ 55% ethanol FE and 80% C_2/2+_ product FE at −1.2 V versus RHE	Dynamical formation of Cu nanoparticles favorable for C–C coupling
Cu(II)‐phthalocyanine^[^ ^99^ ^]^	Purchase commercially	0.5 m KHCO_3_ 66% methane FE at −1.06 V versus RHE with current of 13 mA cm^−2^	Unknown
Cu–Pd_10_Te_3_ catalyst^[^ ^100^ ^]^	Wet impregnation method	0.2 m NaHCO_3_ 92% CO FE at −0.78 V versus RHE	Cu_1_ *^x^* ^+^ moiety facilitated H_2_O molecule adsorption, adsorbed H_2_O stabilized CO_2_ on the Cu_1_^0^ moiety
Cu–CeO_2_‐4% catalyst ^[^ ^29^ ^]^	Wet impregnation method	0.1 m KHCO_3_ ≈58% methane FE at −1.8 V versus RHE	Atomic Cu sites, surrounded multiple oxygen vacancies, and cooperative effect from CeO_2_

Overall, single‐atom Cu catalysts usually exhibit synergistic interactions and the dynamic conversion of active sites during the ECO_2_RR, which complicates the elucidation of the mechanism of synthesis‐directed structural engineering and intermediate modulation. Thus, the interpretation of this mechanism should consider 1) the type of single‐atom Cu catalyst that is stable and capable of undergoing surface reconstruction, 2) the type of surface reconstruction that is reversible, and 3) the significance of the intrinsic instability of the reconstruction.

### 3.6 Surface Modification

Surface modification is one of the most efficient methods for tuning the solution–electrode–gas triple‐phase boundaries of Cu catalysts to facilitate the ECO_2_RR. For this purpose, various surface modifiers including inorganic molecules and atoms as well as organic molecules were studied with precise synthetic processes. These surface modifiers may not only modify the electronic surface and structural geometry of substrates, but also tune the reactant‐ and intermediate‐active site interactions as well as the local electrochemical environment.

Among the inorganic modifiers, the most popular ones are surface‐bonded fluorine, oxygen, hydroxyl, and metal moieties. Ma et al. studied a fluorine‐modified Cu electrocatalyst prepared by the electroreduction of a solvothermally obtained Cu(OH)F compound.^[^ ^101^ ^]^ The solvothermal process enabled the incorporation of OH and F into the Cu components, while the electroreduction selectively removed the OH component to afford F‐modified Cu. The surface F content was measured as ≈6 mol%, and XAS and XPS analyses indicated that the Cu oxidation state was between 0 and +1. The key role of the surface F modifier was the enhancement of *CO adsorption, H_2_O activation to produce hydrogen, and subsequent *CO hydrogenation to facilitate the coupling of *CHO on the Cu surface. Consequently, F‐modified Cu achieved an FE of 80% for C_2/2+_ products at −0.89 V versus RHE, with the current density in the flow cell test reaching 1600 mA cm^−2^. Similar to the F modification, surface hydroxyl modification could stabilize *CO for C–C coupling to generate oxygenated products.^[^ ^102^ ^]^ Interestingly, Cu itself can also be used as an adsorbent to further modify other metal nanoparticles and thus direct the product of ECO_2_RR. A Cu‐modified Au catalyst prepared by underpotential deposition offered tunable CO binding to control the ratio of H_2_ and CO during electrosynthesis.^[^ ^103^ ^]^

**Figure 8:**
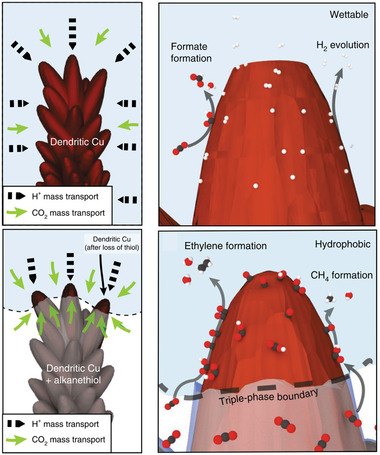
Schematic of the role comparison of hydrophilcity and hydrophobicity in influencing ECO_2_RR relative to hydrogen evolution. Reproduced with permission.^[^ ^104^ ^]^ Copyright 2019, Nature Publishing Group.

Compared to inorganic nonmetal–metal modifiers, organic modifiers allow better design ability and even the physical property tuning of Cu catalysts. For example, the simple immersion of Cu into liquid 1‐octadecanethiol at 60 °C afforded an alkanethiol layer on hierarchically structured Cu dendrites.^[^ ^104^ ^]^ This hydrophobic surface greatly suppressed the HER (as reflected by the corresponding FE drop from 71% to 10%) and promoted the ECO_2_RR (FE values of 56% and 17% achieved for ethylene and ethanol at −1.5 V vs RHE, respectively). The corresponding mechanistic study revealed that the repulsion of the electrolyte from the hydrophobic Cu surface afforded abundant electrolyte–solid–gas triple‐phase boundaries (**Figure** **8**). Therefore, CO_2_ could be rapidly transferred to the reaction surface, which enabled the formation of more *CO for C–C coupling and increased the possibility of *CO protonation to enhance the production of more valuable products. However, another possibility of surface layer decomposition should also be considered in organics‐modified Cu catalysts during ECO_2_RR. Beyond physical property adjustment, electronic modulation and surface geometry control were also demonstrated using dipping into poly(acrylamide) and benzimidazole solutions. A poly(acrylamide)‐modified Cu electrode^[^ ^105^ ^]^ showed enhanced ethylene production with an FE of 26% at −0.96 V versus RHE (cf. FE = 13% without surface modification). This remarkable enhancement was ascribed to the promotional effect of poly(acrylamide) on CO adsorption. Besides, charge transfer from the polymer to Cu facilitated CO activation for dimerization, and the *CO dimer could be further stabilized by the —NH_2_ groups of poly(acrylamide). These combined effects promoted the formation of ethylene. Overall, the surface modification of Cu catalysts with tunable physicochemical properties could be achieved via a simple synthesis. However, the evaluation of surface modifier stability during the ECO_2_RR (desorption, degradation, or conversion) should gain more research attention. Details on surface modification are summarized in **Table** **6**.

**Table 6:** The key synthesis process, and structure–performance insights on various Cu‐based catalysts with surface modification engineering

Electrocatalysts	Synthesis method/process	Electrolyte and ECO_2_RR performance	Primary structure–performance relationship
Fluorine‐modified Cu catalyst^[^ ^101^ ^]^	Solvothermal process and electroreduction	1 m KOH in flow cell 80% C_2/2+_ FE at −0.89 V versus RHE, current:1600 mA cm^−2^	Surface F‐modifier enhanced *CO adsorption, H_2_O activation, and *CO hydrogenation
OH_ad_‐modified Cu electrode^[^ ^102^ ^]^	Electrochemical treatment (pulsed voltammetry)	Phosphate buffer solution (pH = 7.9) Production of oxygenated hydrocarbon	Cooperativity effect between *OH and *CO facilitated decreasing HER, and formation of C—C bonds to generate oxygenated products
Cu‐enriched Au catalyst^[^ ^103^ ^]^	Underpotential deposition of Cu on Au	0.5 m KHCO_3_ Syngas production with current more than 20 mA cm^−2^	Cu modified the surface electronic structure and influenced the *CO binding
Alkanethiols‐coated dendritic Cu^[^ ^104^ ^]^	Electrodeposition and wet impregnation method	0.1 m CsHCO_3_ 56% ethylene FE and 17% ethanol FE at −1.5 V versus RHE	Plastron effect and suppressing HER
Poly(acrylamide)‐modified Cu^[^ ^105^ ^]^	Wet impregnation method	0.1 m NaHCO_3_ 26% ethylene FE at −0.96 V versus RHE	Charge donation to Cu for CO activation, facilitation of *CO adsorption and stabilization of *CO dimer
Benzimidazole‐modified Cu foil^[^ ^106^ ^]^	Wet impregnation method	0.1 m KHCO_3_ ≈77% C_2/2+_ FE at −1.07 V versus RHE	Restriction of H diffusion and highly active H^*δ*+^ from modifier to promote *COOH formation

### 3.7 Nano‐ and Microstructures

Nano‐ and microstructure engineering refers to the nano‐ and microscale synthesis controls over the catalyst structure to form surface‐active locations^[^ ^107^, ^108^, ^109^ ^]^ and allows one to control the local electrocatalytic environment, mainly by limiting the mass transfer toward optimal reactant and intermediate states and electrolyte conditions.

**Figure 9:**
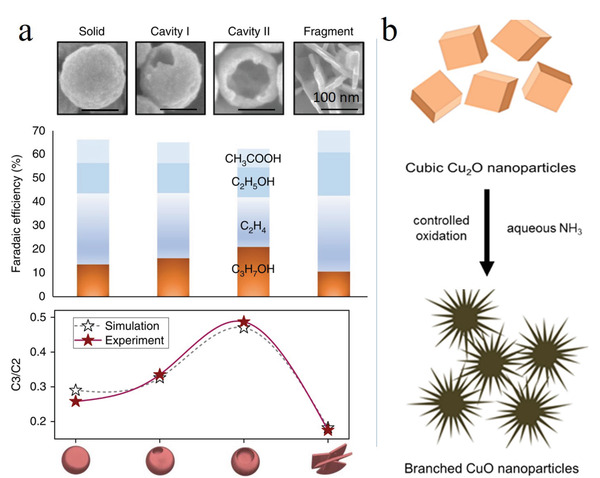
a) Representative SEM images, FE of C3 and C2 products, and the ratio of C3/C2 along with different Cu catalyst morphologies. Adapted with permission.^[^ ^112^ ^]^ Copyright 2018, Nature Publishing Group. b) Schematic of synthesis for branched CuO nanoparticles. Adapted with permission.^[^ ^113^ ^]^ Copyright 2019, American Chemical Society.

A Ag core–porous Cu shell catalyst was prepared by the reduction of AgCl with ascorbic acid followed by the reduction of Cu(NO_3_)_2_ with hydrazine in the presence of poly(ethylene glycol) methyl ether to create a porous structure.^[^ ^110^ ^]^ In this catalyst, Ag was mainly responsible for the production of CO, while the Cu shell with porous channels trapped CO to increase its content. The cascade reaction mechanism featuring the cooperation of both aspects led to the production of C_2/2+_ products. Furthermore, the pore size modulation of Cu catalysts altered the product distribution (e.g., the C_2_H_4_/C_2_H_6_ ratio) because of the influence of the residence time of intermediates (e.g., *CO) in the pores.^[^ ^111^ ^]^ A gentle acidic etching treatment and electroreduction were used to sequentially convert Cu_2_O nanoparticles into Cu_2_O cavities and Cu cavities, respectively. Remarkably, C_3_ alcohols could be produced by catalysts with Cu nanocavity sizes suitable for the stabilization of C_2_ intermediates (**Figure** **9**a).^[^ ^112^ ^]^

The optimization of local pH is an important modulation of the electrocatalytic environment during the ECO_2_RR. As mentioned above, high local pH suppresses the HER and thus increases the selectivity for high‐value‐added products by facilitating multiple PCET processes of the ECO_2_RR. Branched and porous dendritic Cu is effective for the formation of favorable local pH environments. Branched Cu oxide nanoparticles with tunable pore structures and high specific areas were prepared by the slow oxidation of cubic Cu_2_O nanoparticles in aqueous NH_4_OH (Figure 9b).^[^ ^113^ ^]^ The abundant highly active junctions and interfaces allowed this catalyst to achieve an FE of 70% for ethylene production at −1.05 V versus RHE. On the other hand, a nanoporous Cu electrocatalyst was prepared by precipitating Cu(NO_3_)_2_ in an NH_4_OH solution to form Cu(OH)_2_ nanorods, their thermal annealing to form nanoporous CuO, and the in situ electrochemical reduction of this oxide.^[^ ^114^ ^]^ In view of its advantageous surface characteristics, high pH, and fast gas transport, this catalyst achieved an FE of ≈62% for C_2/2+_ products with a current density of 653 mA cm^−2^ at ≈−0.67 V versus RHE in a flow cell test. In general, chemical methods were widely applied for engineering nano‐ and microstructures of Cu with specific design. Recently, a physical process by laser ablation was reported for creation of microstructured Cu,^[^ ^37^ ^]^ which shows great promise for microstructure engineering of the GDE in the application of flow cells. In addition to the microstructure engineering of Cu, the hydrophobic polymer layer can also be engineered with microstructures followed by loading Cu nanoparticles to fabricate GDE,^[^ ^115^ ^]^ resulting in the increased selectivity and partial current for C_2_H_4_ production by 100 and 1.8 times, respectively. Overall, nano‐ and microstructure engineering does not influence the intrinsic properties of Cu active sites but strongly affects ECO_2_RR selectivity due to controls over reactant and intermediate states and the local environment. More importantly, this engineering can be easily designed to synergistically combine with other kinds of structural engineering in synthesis to more significantly promote the selectivity and activity of Cu‐based catalysts. Details on nano‐ and microstructure engineering are summarized in **Table** **7**.

**Table 7:** The key synthesis process, and structure–performance insights on various Cu‐based catalysts with nano‐ and microstructure engineering

Electrocatalysts	Synthesis method/process	Electrolyte and ECO_2_RR performance	Primary structure–performance relationship
Nano porous Cu–Ag alloy^[^ ^109^ ^]^	Electrodeposition with presence of 3,5‐diamino1,2,4‐triazole	0.1 m KHCO_3_ in flow cell 60% C_2_H_4_ FE and 25% C_2_H_5_OH FE at −0.7 V versus RHE, current: ≈300 mA cm^−2^	Enhanced stabilization of Cu_2_O overlayer and optimal availability of CO intermediate with presence of Ag
Porous Cu microspheres^[^ ^108^ ^]^	Electroreduction of Cu_2_O in KI solution	0.05 m KHCO_3_ 78% C_2/2+_ FE at −1.1 V versus RHE	Moderate coordination number (7.7) of Cu in the pore
Open Cu nanocavity structures^[^ ^112^ ^]^	Nucleation and growth of nanocrystals, acid etching to form nanocavity	1 m KOH in flow cell 21% proponol FE at −0.56 V versus RHE with current of ≈7.8 mA cm^−2^ in CO electroreduction	Nanocavity enabled the trapping of C2 intermediates for further reaction
Ag core‐porous Cu shell nanoparticles^[^ ^110^ ^]^	Chemical reduction with presence of poly(ethylene glycol) methyl ether	0.1 m KHCO_3_ ≈5.5*10^−8^ mol s^−1^ g^−1^ for production of C_3_H_8_O at −0.65 V versus RHE	Ag produced CO, and Cu for C–C coupling reaction
Porous 3D Cu skeleton^[^ ^111^ ^]^	Electrochemical deposition	0.5 m NaHCO_3_ 29.1% C_2/2+_ FE at −1.1 V versus RHE	Pores in micrometers to prolong the reaction time
Branched Cu oxide nanoparticles^[^ ^113^ ^]^	Slow oxidation of cubic Cu_2_O in NH_3_ solution	0.1 m KHCO_3_ 70% ethylene FE at −1.05 V versus RHE	Highly active junctions and interfaces, and high local pH
Nanoporous Cu electrocatalyst^[^ ^114^ ^]^	Precipitating Cu salt, thermal annealing, and electroreduction	1 m KOH in flow cell ≈62% C_2/2+_ product FE at ≈−0.67 V with the current of 653 mA cm^−2^	Fast gas transport and high local pH for favorable C–C coupling reaction

To end up, we have discussed some typical works on Cu‐based catalysts, ranging from synthesis to structural engineering (Tables 1, 2, 3, 4, 5, 6, 7), and focused on selectivity control over Cu‐based catalysts to reveal intrinsic structure‐intermediate's interaction‐related insights. To go one step further, one should also correlate synthesis controls with structural engineering and final intermediate modulation with product selectivity, as such correlations can inspire the development of more advanced experimentations toward a new class of selective and efficient Cu‐based electrocatalysts.

### 3.8 Relation of Synthesis, Structural Engineering, and Selectivity

From the above discussion, one can conclude that wet chemical and electrochemical processes are the ones most widely used for the synthesis of Cu‐based electrocatalysts (**Table** **8**). These methods involve multiple processes (e.g., reduction, deposition, etching, adsorption, and mixing) with controlled rates and thus show good controllability and variability in reaction types and synthesis conditions. Solvothermal methods, as a specific wet chemical process, are normally performed in subcritical or supercritical conditions to increase the reactivity of reactants, facilitating the formation of Cu‐based compounds with specific structures relying on different reactant diffusion mechanisms. Thermal treatment is independent from the wet chemical process, and could be the necessary step of complicated syntheses and also the primary step of the fabrication of heterostructured and single‐atom Cu catalysts. Among these synthetic protocols, the choice for single‐atom Cu catalysts is strongly influenced by substrate nature, e.g., high‐temperature thermal treatment is recommended for carbon‐based substrates (such as N‐doped C or C_3_N_4_), whereas wet chemical processes are preferred for metal substrates. For the synthesis of single‐atom Cu catalysts in high yield, the newly developed Cu–Li amalgam method holds great promise;^[^ ^97^ ^]^ while for the synthesis of heterostructured Cu materials, the methods featuring physical combination based on mixing, sputtering, or deposition are preferred. In particular, synergistic effects allow one to achieve high ECO_2_RR selectivity and activity in Cu‐based electrocatalysts, the synthesis of which thus requires multiple structural engineering approaches and effective combinations of different synthetic methods and processes. Among these alternative methods/processes, wet chemical processing offers flexibility and scalability and should therefore be considered for the synthesis of Cu‐based catalysts.

**Table 8:** Statistical example number (red dots) on the primary synthesis methods and processes from Tables 1, 2, 3, 4, 5, 6, 7 resulting in specific structural engineering in Cu‐based catalysts. Wet chemical process includes chemical reduction in aqueous solution and organic solvent, wet impregnation process, and chemical etching under standard atmosphere pressure. Electrochemical process involves electrodeposition, electroreduction, and electrochemical activation. Thermal treatment includes pyrolysis and annealing. Physical method treatment refers to physically melting, physical vapor deposition, physical mixing, and sputtering processes

	Wet chemical process	Electrochemical process	Solvothermal process	Themal treatment	Physical process	Amalgamated Cu–Li method
Alloy	●●●	●●			●●	
Compound	●	●	●●			
Doping	●	●●				
Defect		●				
Crystal facet	●●●●	●●●●●●				
Amorphization	●				●	
Heterostructure	●●●●●	●●●●●		●●	●●●●●●	
Single atom	●●			●●●●		●
Surface modification	●●●	●●●●				
Nano–microstructure	●●●	●●●		●		

**Figure 10:**
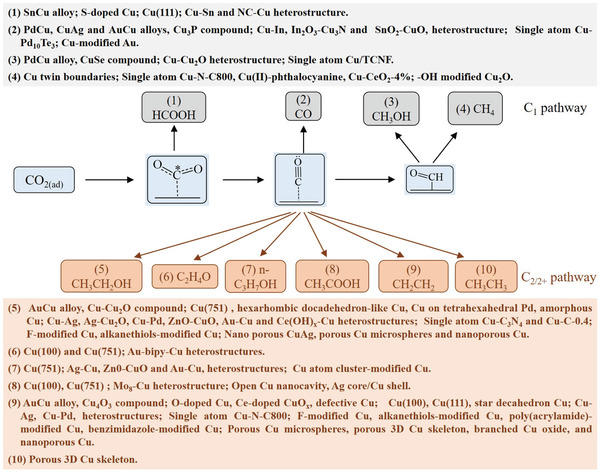
Summary on the main product from ECO_2_RR on different engineered Cu structures.

**Figure** **10** summarizes the main ECO_2_RR products in association with the engineered structure characteristics of Cu‐based catalysts, revealing that ethanol and ethylene are common products, and the formation of acetaldehyde and ethane has been rarely reported thus far. The ECO_2_RR on most Cu‐based catalysts usually affords a mixture of different products rather than a single product, with the exact composition mainly determined by the modulation of intermediates (among other factors). To gain a deeper understanding of the intrinsic mechanisms, we have further summarized intermediate modulations together with the additional effects on different Cu‐based catalysts in terms of structural engineering categories (**Table** **9**). As the modulations over the *CO intermediate have received the most attention, it is rational to suggest that this modulation is of key importance for different structural engineering types. Specifically, the modulation refers to three aspects, namely, *CO generation, stabilization, and the subsequent reaction, e.g., CO–CO coupling. Different structural engineering approaches further affect the adsorption and reactivity of other intermediates (e.g., *CH_2_CHO, *CHO, *OCH_2_, *H, and *CH_3_) and thus, in combination with the additional effects (e.g., HER suppression, oxophilicity reduction, electron transfer promotion, and local pH) to ultimately determine ECO_2_RR selectivity and activity. Density functional calculation is a powerful tool for the determination of reaction pathways but too tedious to perform. Therefore, the prediction of some key intermediate modulations as well as that of other synergistic effects for an engineering structure of choice could aid the screening for new Cu‐based electrocatalysts with high selectivity and activity. Table 9 provides some related information for such predictions.

**Table 9:** Summary on intermediate modulation and additional effects on different engineered Cu structures

	Enhanced intermediate adsorption^a^/reactivity^b^	Suppressing intermediate adsorption^c^/reactivity^b^	Additional effects
Alloying/compounding engineering	PdCu (*CO/*CHO)^a^, AuCu (CH_2_CHO*, CH_3_CHO*, and CH_3_CH_2_O*)^a^, SnCu (CO_2_•^−^ and HCOO• generation)^b^, CuSe (*COOH, *CO, *CHO, *OCH_2_, and *OCH_3_ generation)^b^	PdCu (*CO protonation)^b^, CuAg (*H and O‐containing intermediates)^c^	PdCu (S‐HER), CuAg(reduced oxophilicity), SnCu (S‐HER)
Doping and defect engineering	S‐doped Cu (*OCHO)^a^, O‐doped Cu (*CO)^a^, Ce‐doped Cu (*CH_2_–*CH_2_ coupling, *C–*CO coupling, *CH_2_ protonation)^b^		Truncated‐octahedral‐Cu_2_O (enhanced charge transfer)
Crystal face engineering and amorphization	Cu(111) (formate formation)^b^, Cu(100) (*CO)^a^, Cu nanowires (*CO)^a^, Cu twin boundaries (*CO protonation)^b^, star decahedron Cu (*CHO generation)^b^; amorphous Cu (CO_2_)^a^	Hexarhombic docadehedron‐like Cu (*O)^c^	
Heterostructure engineering	Cu–Ag (*CO generation)^b^, Ag–Cu_2_O (*CO generation)^b^, Au‐bipy‐Cu (*CO generation)^b^ and (*CO_2_ protonation)^a,b^, Cu–Cu_2_O (*CO)^a^; ZnO–CuO (*CO, *CH_3_)^a^, Cu–In (*COOH)^a^, Cu–Sn (*COOH generation)^b^, Mo_8_–Cu (*CH_3_ generation)^b^, NC–Cu (*CO)^a^, Au–Cu (*CO generation)^b^, Ce(OH)*_x_*–Cu (*HCCOH hydrogenation)^b^	Cu–Cu_2_O (*H)^c^, Cu–In (*H)^c^	Ag–Cu (geometric effect), Cu–Pd (geometric effect), NC–Cu (increased conductivity)
Single atom engineering	CuSAs/TCNFs (*CO)^a^, Cu–N–C (*CO protonation)^b^, Cu–C_3_N_4_ (C‐/O‐terminated intermediate)^a^, Cu–Pd_10_Te_3_ (*H_2_O)^a^		Cu/C (formation of Cu cluster), Cu–N–C (formation of Cu nanoparticle)
Surface modification	F‐modified Cu (*CO)^a^ and (H_2_O activation, *CO hydrogenation)^b^, —OH‐modified Cu_2_O (*COOH)^a^, Cu atom‐modified Cu (*CO)^a^, Cu‐modified Au (*CO)^a^, poly(acrylamide)‐modified Cu (*CO activation)^a,b^, benzimidazole‐modified Cu (*COOH formation)^b^	Benzimidazole‐modified Cu (H diffusion)^b^	—OH‐modified Cu_2_O (S‐HER), —OH‐modified Cu (S‐HER), alkanethiols‐coated Cu (plastron effect and S‐HER)
Nano/microstructure engineering	Open Cu nanocavity (C2 intermediate trapping)^a^, Ag core–porous Cu shell (CO generation and coupling)^a,b^		Porous 3D Cu skeleton (prolonged reaction time), branched Cu oxide nanoparticle (high local pH), nanoporous Cu (fast gas transport, high local pH)

Note: S‐HER represents suppressing HER. Intermediate reactivity includes its generation, protonation, and so on.

## 4 Challenges and Outlook

This review offers an integral Cu‐based material design framework for ECO_2_RR, based on synthetic methodology‐directed structure design and the resultant intermediate modulation toward boosting ECO_2_RR performance. Development of Cu‐based catalysts toward high selectivity/activity relies highly on optimal structural engineering design, which is influenced and facilitated by the development of more efficient synthetic chemistry. The current opportunities, challenges, and perspectives of the development of more advanced synthetic chemistry toward Cu catalysts for the highly selective ECO_2_RR are elaborated as follows.

The *CO intermediate modulation is revealed to be crucial toward the production of high‐value‐added products. Therefore, the goal for synthetic chemistry design on Cu should be guided to alloying with Au, Ag, and Zn; compounding with Se, combination with NC, doping with O and Ce, fabrication of Cu(100) surfaces, nanowires, and twin boundaries, downsizing Cu to single atoms on carbon substrates, modification of Cu particles with F, Cu, and poly(acrylamide), and the construction of nanoporous structures. All of these structural engineering may facilitate the *CO intermediate generation, stabilization, and/or dimerization, and thus the production of high‐value‐added products. In addition to this, such synthetic chemistry design also provides the basis for the further modulation over other intermediates such as *CH_2_CHO, *CHO, *OCH_2_, *H, and *CH_3_ and the combination of additional effects (HER suppression, oxophilicity reduction, electron transfer promotion, and local pH control), to determine the ultimate ECO_2_RR selectivity and activity. In particular, the occurrence of site reconstruction of Cu‐based catalysts during ECO_2_RR suggests that earlier‐stage synthetic chemistry does not always determine the final performance. For example, the conversion from single‐atom Cu to Cu clusters and nanoparticles alters intermediate generation, stabilization, and dimerization, thus necessitating the redefinition of the promotion mechanisms. Therefore, the synthetic chemistry should be applied throughout the course of the common synthetic and ECO_2_RR processes.

Some promising synthesis‐controlled structural characteristics can be highlighted: Cu‐based catalysts (Cu–Ag, Cu_2_O, and metallic Cu) with porous structures may enhance ethanol and ethylene production compared to the counterparts without porous structure. ECO_2_RR on heterostructured Cu catalysts could derive different products, depending on the controls over interface species, electronic structure of active sites, and the synergy of the two heterostructure components. Defect engineering and amorphization of Cu‐based ECO_2_RR catalysts remain much research room, with one of the research focuses on the development of the more effective synthesis methods and processes. In particular, synergistic effects could promote high‐value‐added product formation via tandem and/or sequential catalysis mechanisms. Nano‐ and microstructure engineering alters the local intermediate content and/or electrochemical environment, and could thus synergistically tune ECO_2_RR performance. To realize synergistic effects, multiple structural engineering methods are necessary. For the synthesis of multiple structure‐engineered Cu‐based catalysts, one is strongly advised to develop combined methods or novel approaches, e.g., synthesis of a facet‐guided heterostructure Cu catalyst via a wet chemical method followed by physical vapor deposition.

With the classification and correlation of various intermediate generation, stabilization, and dimerization, structural engineering, and corresponding synthesis methods/processes, machine‐learning algorithms can be further adapted to predict promising structures, combination forms, and synthetic methodologies to promote the experimental development of Cu‐based catalysts. The coupling between laboratory investigations and practical applications was enhanced by the recent progress in the optimization of nanostructured electrodes, alkaline electrolytes, flow cells, and operation conditions (e.g., pressure, temperature, and flow rate). To further boost the practical applicability of advanced synthetic chemistry, two important features should be considered, namely, the use of cost‐efficient feedstocks and the avoidance of tedious synthetic steps.

Despite the significant progress in this field, the development of more advanced experimentations toward the design of Cu catalysts for efficient ECO_2_RR remains challenging. The combination of multiscale structure design and synthesis methodologies holds great promise for the provision of a predictable platform to tackle the ECO_2_RR bottleneck. With the continuous research and development of synthetic chemistry, the ECO_2_RR promoted by Cu‐based catalysts is expected to demonstrate an influential role in addressing the energy and environmental issues in the future.

## Conflict of Interest

The authors declare no conflict of interest.
